# Association Between Care Management and Outcomes Among Patients With Complex Needs in Medicare Accountable Care Organizations

**DOI:** 10.1001/jamanetworkopen.2019.6939

**Published:** 2019-07-12

**Authors:** Mariétou H. Ouayogodé, Alexander J. Mainor, Ellen Meara, Julie P. W. Bynum, Carrie H. Colla

**Affiliations:** 1 The Dartmouth Institute for Health Policy & Clinical Practice, Geisel School of Medicine at Dartmouth, Lebanon, New Hampshire; 2 The National Bureau of Economic Research, Cambridge, Massachusetts; 3 Department of Internal Medicine, University of Michigan, Ann Arbor

## Abstract

**Importance:**

People with complex needs account for a disproportionate amount of Medicare spending, partially because of fragmented care delivered across multiple practitioners and settings. Accountable care organization (ACO) contracts give practitioners incentives to improve care coordination to the extent that coordination initiatives reduce total spending or improve quality.

**Objective:**

To assess the association between ACO-reported care management and coordination activities and quality, utilization, spending, and health care system interactions in older adults with complex needs.

**Design, Setting, and Participants:**

In this cross-sectional study, survey information on care management and coordination processes from 244 Medicare Shared Savings Program ACOs in the 2017-2018 National Survey of ACOs (of 351 Medicare ACO respondents; response rate, 69%) conducted from July 20, 2017, to February 15, 2018, was linked to 2016 Medicare administrative claims data. Medicare beneficiaries 66 years or older who were defined as having complex needs because of frailty or 2 or more chronic conditions associated with high costs and clinical need were included.

**Exposures:**

Beneficiary attribution to ACO reporting comprehensive (top tertile) care management and coordination activities.

**Main Outcomes and Measures:**

All-cause prevention quality indicator admissions, 30-day all-cause readmissions, acute care and critical access hospital admissions, evaluation and management visits in ambulatory settings, inpatient days, emergency department visits, total spending, post–acute care spending, health care contact days, and continuity of care (from Medicare claims).

**Results:**

Among 1 402 582 Medicare beneficiaries with complex conditions, the mean (SD) age was 78 (8.0) years and 55.1% were female. Compared with beneficiaries assigned to ACOs in the bottom tertile of care management and coordination activities, those assigned to ACOs in the top tertile had identical median prevention quality indicator admissions and 30-day all-cause readmissions (0 per beneficiary across all tertiles), hospitalization and emergency department visits (1.0 per beneficiary in bottom and top tertiles), evaluation and management visits in ambulatory settings (14.0 per beneficiary [interquartile range (IQR), 8.0-21.0] in both tertiles), longer median inpatient days (11.0 [IQR, 4.0-33.0] vs 10.0 [IQR, 4.0-32.0]), higher median annual spending ($14 350 [IQR, $4876-$36 119] vs $14 229 [IQR, $4805-$36 268]), lower median health care contact days (28.0 [IQR, 17.0-44.0] vs 29.0 [IQR, 18.0-45.0]), and lower continuity-of-care index (0.12 [IQR, 0.08-0.20] vs 0.13 [IQR, 0.08-0.21]). Accounting for within-patient correlation, quality, utilization, and spending outcomes among patients with complex needs attributed to ACOs were not statistically different comparing the top vs bottom tertile of care management and coordination activities.

**Conclusions and Relevance:**

The ACO self-reports of care management and coordination capacity were not associated with differences in spending or measured outcomes for patients with complex needs. Future efforts to care for patients with complex needs should assess whether strategies found to be effective in other settings are being used, and if so, why they fail to meet expectations.

## Key Points

**Question:**

What is the association between care management and outcomes among patients with complex needs in Medicare accountable care organizations?

**Findings:**

In this cross-sectional analysis of 1 402 582 US Medicare beneficiaries who had frailty or multiple chronic conditions, beneficiaries attributed to accountable care organizations in the top vs bottom tertile of reported care management and coordination activities did not have statistically different quality, utilization, spending, or health care system interaction measures.

**Meaning:**

Accountable care organization–reported care management and coordination activities were not associated with improved outcomes among patients with complex needs.

## Teaser

This cross-sectional study uses data from the US National Survey of Accountable Care Organizations to assess the association between accountable care organization–reported care management and coordination activities and outcomes in older adults with complex needs.

## Introduction

For older adults who are frail or have multiple chronic conditions (ie, patients with complex needs), fragmentation of care across settings and practitioners can lead to difficulty navigating the health care system, unnecessary spending, and gaps in care quality. Consistent with this challenge, a small proportion of patients (5%) account for more than half of medical spending.^1^ Accountable care organizations (ACOs) are redesigning care for these patients by building robust care management and care coordination strategies based on the idea that focusing on these patients is likely to yield the greatest cost savings.^2^ Moreover, care management and coordination activities may represent important strategies for achieving savings and quality standards under ACO programs.^3^

Qualitative research has found that many ACO care management and coordination programs are focused on transforming primary care through increased team-based care, strengthening practice-based care management, and developing new roles and activities that span care settings.^2^ However, the evidence for the effectiveness of care management and coordination use in ACOs to date is mixed. One study^3^ of a Pioneer ACO evaluated its care management program and found consistent reductions in spending and utilization over time, with a strong association between length of time in the program and results. Another article^4^ studied unplanned hospitalizations for select chronic conditions and found no association between ACO participation and reduction in hospital use, concluding that care coordination efforts have minimal associations with hospitalization of patients with chronic diseases. However, this work aggregated all ACOs regardless of how comprehensive their care management and coordination activities were, potentially obscuring significant savings in ACOs where care management and coordination was prioritized.

One of the challenges for the research in this area is defining and measuring what is meant by care management and care coordination activities. The notion of care management and coordination is fraught with disagreement over the importance of individual components and interventions.^5^ Previous research^2^ has distinguished care management from care coordination activities in ACOs. Both care management, which refers to programs and systems that aim to help manage patients’ health and medical conditions, and care coordination, which refers to activities to integrate care across practitioners, require organizational investments because the associated services typically cross boundaries of professions and settings. Despite ongoing investment in care management and coordination programs by ACOs,^6,7^ there is little empirical research examining the heterogeneity across ACOs in these activities or the association of those activities with outcomes.

By using detailed, survey-based information collected from ACOs participating in the Medicare Shared Savings Program (MSSP), we described and quantified the care management and coordination efforts implemented by ACOs and assessed whether more comprehensive efforts were associated with better outcomes (quality, utilization, and spending) for older adults with complex needs in Medicare.

## Methods

For this cross-sectional study, we used the 2017-2018 National Survey of ACOs (NSACO) to evaluate care management and coordination strategies reported as being used by MSSP ACOs to provide care to patients with complex needs. We used the Centers for Medicare & Medicaid Services MSSP attribution method to assign beneficiaries to the provider organization with the greatest allowed charges for eligible primary care services.^8^ This study was reviewed and approved by the institutional review board of Dartmouth College. Written informed consent was obtained from survey respondents, and data were deidentified. The study was conducted in accordance with the Strengthening the Reporting of Observational Studies in Epidemiology (STROBE) reporting guideline.^9^

### NSACO Wave 4 Survey Data

We gathered data on MSSPs’ reported care management and coordination activities from the 2017-2018 NSACO. This survey was conducted from July 20, 2017, to February 15, 2018. We identified Medicare ACOs for the survey from Centers for Medicare & Medicaid Services data. The response rate for Medicare ACOs was 69%. The ACO-affiliated leadership personnel (eg, executive directors, vice presidents, chief executive officers, medical directors, chief operating officers, director, and account managers) responded to the survey either online or via a paper survey. The survey included questions related to ACO organizational characteristics and strategies used by ACOs to meet contract goals, including several questions on care management and coordination programs and activities.

The NSACO contained questions on care management and care coordination strategies relevant to patients with complex needs, including processes and programs for predictive risk stratification and segmentation, chronic care management, active patient involvement in decisions that involved their care and self-management of their condition, and care transition processes and services.^10^ These topics, drawn from 6 multipart survey questions, resulted in 12 variables for inclusion (eTable 1 in the Supplement). We computed an index score—referred to as care management and coordination index—that measured reported care management and coordination activities for each ACO and their ability to simultaneously implement care management and coordination processes. We assigned each response discrete point values that ranged from 0 for scores of 1 to 3, 1 for scores of 4 to 6, and 2 for scores of 7 to 9 on a 9-point Likert scale (4 questions, with 1 indicating no processes or programs in place and 9 indicating comprehensive processes or programs in place), and we used values of 1 or 0 for binary yes-or-no response options, resulting in a total of 0 to 16 points maximum. The care management and coordination index was similar to a factor analysis–based index and easier to interpret; the correlation between the first factor score and our computed point-based index was 0.94 (eTable 1 in the Supplement). The ACOs were ranked into tertiles based on their level of activity captured by the care management and coordination index. The main exposure variable was whether a beneficiary with complex needs (with frailty or multiple chronic conditions) was attributed to an ACO with a high reported care management and coordination index.

### Patient Population

We used Parts A and B 2016 Medicare fee-for-service claims to capture care patterns, diagnoses, use, and spending and the beneficiary summary file to capture patient demographic characteristics, enrollment status, and date of death. We used beneficiaries’ zip codes to identify whether they lived in high-poverty census tracts (≥20% of residents below the federal poverty level)^11^ and to determine hospital referral regions of residence.

To identify patients with complex needs in Medicare claims, we identified eligible Medicare beneficiaries who were 66 years or older, had full Parts A and B fee-for-service coverage, and were living in the 50 states or the District of Columbia, and we limited our sample to 2 groups: frail, older patients and patients with multimorbidities. Following the claims-based frailty indicators defined by Kim and Schneeweiss^12^ and Joynt et al,^13^ we created a cohort of frail, older patients (eTable 2 in the Supplement). We used *International Statistical Classification of Diseases and Related Health Problems, Tenth Revision* codes to identify beneficiaries in the multimorbidity cohort, which included those with at least 2 of a list of 18 chronic conditions^14^ selected for their chronic nature and association with mortality and costs (eTable 3 in the Supplement).^15^

### Variable Descriptions

We reported beneficiary demographic characteristics (age in years, sex, race/ethnicity, and socioeconomic status) and dual eligibility for Medicaid. We identified nursing home residents using Medicare Parts A and B claims if they had eligible place of service and *Common Procedural Terminology* codes defining use of a nursing facility (eTable 4 in the Supplement).^16^ For this covariate, we did not distinguish between short-term and long-term stay residents. For each beneficiary, we presented the number of reported hierarchical condition categories in 2016.^15^

The primary outcomes of interest included quality of care, health care utilization, spending, and interactions with the health care system for patients with complex needs. We measured quality with annual measures for the number of all-cause prevention quality indicator admissions^17^ and 30-day all-cause readmissions. We measured health care utilization by examining acute care or critical access hospital admissions, evaluation and management (E&M) visits in ambulatory settings, inpatient days, and emergency department (ED) visits not leading to admission. We measured spending with annual total spending and computed beneficiary total post–acute care spending. Interactions with the health care system were measured with health care contact days, defined as the number of days per year in an inpatient setting or with a practitioner visit, procedure, imaging study, or laboratory test in an ambulatory setting^18^ and, for patients with at least 4 ambulatory visits, a continuity-of-care index calculated as a Herfindahl-Hirschman index for patients. The continuity-of-care index is higher for patients getting more of their care from fewer practitioners, with a value of 1 if all care is received by a single practitioner.^19^ We also reported 1-year mortality status among beneficiaries.

### Statistical Analysis

We compared characteristics among Medicare enrollees with complex needs attributed to ACOs across the care management and coordination index using multivariate regression models. We modeled beneficiary outcomes as functions of the beneficiary-attributed ACO–reported care management and coordination index by tertile, controlling for demographic characteristics, dual eligibility for Medicaid, nursing home residency, frailty, and multiple chronic condition measures. Least squares regression models were used for all outcome measures, and heteroscedasticity-robust SEs were clustered at the ACO level. In sensitivity analyses, we restricted the sample to include beneficiaries with frailty and those with at least 3 of the 18 selected chronic conditions. We also reestimated our initial multivariate regression models and controlled for geography measured by census regions. Statistical tests conducted in this analysis were 2-sided, with *P* < .05 considered to be statistically significant.

## Results

The sample consisted of 244 MSSP ACOs with NSACO survey data on care management and coordination processes. A total of 1 402 582 Medicare beneficiaries with complex conditions (mean [SD] age, 78 [8.0] years; 55.1% female) were studied. Characteristics of beneficiaries attributed to the survey respondents (n = 1 402 582) were similar overall to those attributed to nonrespondents (n = 937 557) except for differences in the proportions of females (55.1% among respondents vs 55.5% among nonrespondents) (eTable 5 in the Supplement), individuals with nonwhite race/ethnicity (13.9% vs 20.6%), beneficiaries living in high-poverty census tracts (17.0% vs 18.3%), nursing home residents (29.6% vs 28.5%), and dually eligible beneficiaries (18.1% vs 21.6%).

The ACOs were grouped into tertiles of the care management and coordination index score (84 in the bottom tertile, 97 in the middle tertile, and 63 in the top tertile) (Table 1), which was skewed to the right and ranged from 2 (lowest intensity) to 16 (highest intensity). The ACOs reported considerable variation in their care management and transition activities, even among those most commonly provided. More than 48 ACOs (76.2%) in the highest care management and coordination index tertile reported that they segmented high-risk patients, dividing them into groups based on common care needs, compared with only 42 (50.0%) in the lowest tertile. Other care management activities were largely similar among ACOs within tertiles, with chronic care management processes being the most comprehensive (median, 8.0 [interquartile range (IQR), 7.0-9.0] for the highest tertile and 6.0 [IQR, 5.0-7.0]) and shared decision-making process being the least (median, 7.0 [IQR, 6.0-8.0] for the highest tertile and 5.0 [IQR, 3.0-6.0] for the lowest tertile on a 9-point scale). Among potential strategies to reduce the risk of readmission for hospitalized patients undergoing a care transition to home or post–acute care, more than 90% of ACOs in the highest tertile of the care management and coordination index reported providing medication reconciliation (58 [92.1%]), transmitting discharge summaries to practitioners accepting care of the patients (58 [92.1%]), having standardized processes in place to ensure timely follow-up with primary and specialty care (61 [96.8%]), and following up via telephone within 72 hours of discharge (60 [95.2%]). In contrast, ACOs reported that the strategy adopted least was in-home follow-up within 72 hours of discharge across each tertile (24 [38.1%] in the highest tertile vs 2 [2.4%] in the lowest tertile). Overall use of patient navigators and care managers for most or all patients varied widely across tertiles (highest tertile: 57 [90.5%]; lowest tertile, 17 [20.2%]), as did the use of care managers or health coaches after discharge (highest tertile, 59 [93.7%]; lowest tertile, 13 [15.5%]).

**Table 1.** Processes That Contribute to Index of Care Management and Coordination Activities by Index Tertile: NSACO Wave 4 Respondents Linking to Older Adult Medicare Beneficiaries With Complex Needs

Characteristic^a^	ACOs With MSSP Contract			*P* Value
	First (Lowest) Tertile (n = 84)	Second (Middle) Tertile (n = 97)	Third (Highest) Tertile (n = 63)	
Care management and coordination index, median (IQR)	7.0 (6.0-9.0)	11.0 (10.0-12.0)	14.0 (13.0-15.0)	<.001
Care management activities				
Segmentation of high-risk patients, No. (%)	42 (50.0)	59 (60.8)	48 (76.2)	.005
Predictive risk stratification ability score, scale 1-9, median (IQR)	5.0 (3.5-6.5)	6.0 (5.0-7.0)	8.0 (6.0-9.0)	<.001
Chronic care management score, scale 1-9, median (IQR)	6.0 (5.0-7.0)	7.0 (6.0-8.0)	8.0 (7.0-9.0)	<.001
Shared decision making score, scale 1-9, median (IQR)	5.0 (3.0-6.0)	6.0 (5.0-6.0)	7.0 (6.0-8.0)	<.001
Care transition activities				
Care transitions score, scale 1-9 scale, median (IQR)	5.0 (4.0-6.0)	6.0 (6.0-7.0)	8.0 (7.0-8.0)	<.001
Medication reconciliation, No. (%) with most or all patients	44 (52.4)	85 (87.6)	58 (92.1)	<.001
Use of patient navigator or care manager, No. (%) with most or all patients	17 (20.2)	42 (43.3)	57 (90.5)	<.001
Discharge summaries transmitted to practitioners, No. (%) with most or all patients	31 (36.9)	86 (88.7)	58 (92.1)	<.001
Standardized process in place to ensure timely follow-up with primary or specialty care, No. (%) with most or all patients	34 (40.5)	89 (91.8)	61 (96.8)	<.001
Telephone follow-up within 72 h of discharge, No. (%) with most or all patients	33 (39.3)	91 (93.8)	60 (95.2)	<.001
Use of care manager or health coach after discharge, No. (%) with most or all patients	13 (15.5)	57 (58.8)	59 (93.7)	<.001
In-home follow-up within 72 h of discharge, No. (%) with most or all patients	2 (2.4)	13 (13.4)	24 (38.1)	<.001

Abbreviations: ACO, accountable care organization; IQR, interquartile range; MSSP, Medicare Shared Savings Program; NSACO, National Survey of Accountable Care Organizations.

^a^ Information on the scales is given in the NSACO Wave 4 Survey Data subsection of the Methods section.

Patients with complex needs (1 402 582 overall, with 445 829 in the bottom tertile, 566 633 in the middle tertile, and 390 120 in the top tertile of the care management and coordination index) represented 29.5% of all MSSP-attributed Medicare beneficiaries 66 years or older. There was overlap among categories; more than 14% of the sample with complex needs met criteria for both frailty and 2 or more chronic conditions. The ACO-attributed beneficiaries with complex needs were similar across tertiles of the care management and coordination index with a few exceptions (Table 2). Patients with complex needs had a median number of hierarchical condition categories of 3.0 (IQR, 2.0-5.0) (all tertiles). Comparing patients attributed to ACOs in the top vs bottom tertiles, patients in the top tertile were less likely to have nonwhite race/ethnicity (13.1% vs 15.2%), live in a high-poverty census tract (16.5% vs 16.8%), and have dual eligibility for Medicaid (17.6% vs 18.1%) but were more likely to reside in a nursing home (30.7% vs 29.5%).

**Table 2.** Descriptive Characteristics of Fee-for-Service Medicare Beneficiaries With Complex Needs Attributed to MSSP ACOs, 2016^a^

Characteristic	ACOs With MSSP Contract (NSACO Respondents Only) by Care Management and Coordination Index^b^			*P* Value
	First (Lowest) Tertile (n = 445 829)	Second (Middle) Tertile (n = 566 633)	Third (Highest) Tertile (n = 390 120)	
Cohort				
Frail and older^c^	99 642 (22.3)	116 854 (20.6)	84 980 (21.8)	<.001
Multimorbidities^d^	412 670 (92.6)	527 794 (93.1)	363 081 (93.1)	<.001
Frail, older, and multimorbidities^c^^,^^d^	66 483 (14.9)	78 015 (13.8)	57 941 (14.9)	<.001
Demographic characteristic				
Age, mean (SD), y	78.2 (8.0)	78.2 (8.0)	78.3 (7.9)	<.001
Female sex	246 466 (55.3)	312 332 (55.1)	214 304 (54.9)	.006
Race/ethnicity^e^				
Non-Hispanic white	377 889 (84.8)	491 018 (86.7)	338 851 (86.9)	<.001
Black	33 548 (7.5)	42 189 (7.4)	29 447 (7.5)	.13
Hispanic	21 396 (4.8)	18 313 (3.2)	12 724 (3.3)	<.001
Asian/Pacific Islander	6586 (1.5)	7274 (1.3)	4102 (1.1)	<.001
Other	6410 (1.4)	7839 (1.4)	4996 (1.3)	<.001
Lives in high-poverty (>20%) census tract	74 896 (16.8)	99 625 (17.6)	64 466 (16.5)	<.001
Dual Medicare and Medicaid status	80 737 (18.1)	105 772 (18.7)	68 735 (17.6)	<.001
Nursing home resident	131 396 (29.5)	164 164 (29.0)	119 778 (30.7)	<.001
Clinical condition history				
No. of hierarchical condition categories, median (IQR)	3.0 (2.0-5.0)	3.0 (2.0-5.0)	3.0 (2.0-5.0)	<.001
Mortality, No. of deaths recorded in 2016	53 236 (11.9)	68 727 (12.1)	48 011 (12.3)	<.001
Outcome variables				
Quality of care per beneficiary, median (range)				
All-cause PQI admissions	0.0 (0.0-13.0)	0.0 (0.0-13.0)	0.0 (0.0-53.0)	<.001
All-cause 30-d readmissions^f^	0.0 (0.0-4.0)	0.0 (0.0-5.0)	0.0 (0.0-4.0)	.05
Health care utilization, median (IQR), per beneficiary				
E&M visits in ambulatory settings	14.0 (8.0-21.0)	13.0 (8.0-20.0)	14.0 (8.0-21.0)	<.001
Acute care or critical access hospital admissions	1.0 (0.0-1.0)	0.0 (0.0-1.0)	1.0 (0.0-1.0)	<.001
Inpatient days^f^	10.0 (4.0-32.0)	10.0 (3.0-30.0)	11.0 (4.0-33.0)	<.001
ED visits	1.0 (0.0-2.0)	1.0 (0.0-2.0)	1.0 (0.0-2.0)	.06
Medicare spending, median (IQR), $				
Total^g^	14 229 (4805-36 268)	14 036 (4766-35 278)	14 350 (4876-36 119)	<.001
PAC^h^	0 (0-5320)	0 (0-4966)	0 (0-5744)	<.001
Interactions with the health care system, median (IQR)				
Health care system contact days	29.0 (18.0-45.0)	27.0 (17.0-43.0)	28.0 (17.0-44.0)	<.001
Continuity-of-care index^i^	0.13 (0.08-0.21)	0.12 (0.08-0.20)	0.12 (0.08-0.20)	<.001

Abbreviations: ACO, accountable care organization; E&M, evaluation and management; ED, emergency department; IQR, interquartile range; MSSP, Medicare Shared Savings Program; NSACO, National Survey of Accountable Care Organizations; PAC, post–acute care; PQI, prevention quality indicator.

^a^ Includes beneficiaries 66 years or older.

^b^ Data are presented as number (percentage) of ACOs unless otherwise indicated.

^c^ Frailty indicators included abnormality of gait, malnutrition or abnormal loss of weight and underweight, adult failure to thrive, cachexia, debility, fall, muscular wasting and disuse atrophy, muscle weakness, decubitus ulcer of skin or pressure ulcer, senility without mention of psychosis, malaise and fatigue, durable medical equipment use, and nursing or personal care services.

^d^ Selected chronic conditions included coronary artery disease, cancer, connective tissue disorders, congestive heart failure, diabetes, dementia, chronic obstructive pulmonary disease, hematologic or thrombotic disease, HIV infection or AIDS, immune disease, liver disease, Parkinson or Huntington disease, paralysis, peripheral vascular disease, renal disease, cerebral hemorrhage or stroke, severe mental illness, and substance use disorder.

^e^ Race/ethnicity percentages may not sum to 100% because of rounding.

^f^ Inpatient days were only computed for patients with observable inpatient admissions. Readmissions were only reported for beneficiaries with nonzero inpatient days.

^g^ Total spending included Medicare Provider Analysis and Review (inpatient), carrier (physician or supplier), outpatient facility, hospice, durable medical equipment, and home health.

^h^ Post–acute care spending included settings such as skilled nursing facilities, home health agency, inpatient rehabilitation facility, long-term care hospital, outpatient rehabilitation facility, and comprehensive outpatient rehabilitation facility.

^i^ Continuity of care indexes were only calculated for a subset of beneficiaries with 4 or more visits in 2016.

Unadjusted outcomes in patients with complex needs varied slightly when examined across care management and coordination index tertiles. Compared with beneficiaries assigned to ACOs in the bottom tertile of care management and coordination activities, those assigned to ACOs in the top tertile had identical median prevention quality indicator admissions and 30-day all-cause readmissions (0 per beneficiary across all tertiles), hospitalization and emergency department visits (1.0 per beneficiary in bottom and top tertiles), evaluation and management visits in ambulatory settings (14.0 per beneficiary [interquartile range (IQR), 8.0-21.0] in both tertiles), longer median inpatient days (11.0 [IQR, 4.0-33.0] vs 10.0 [IQR, 4.0-32.0]), higher median annual spending ($14 350 [IQR, $4876-$36 119] vs $14 229 [IQR, $4805-$36 268]), lower median health care contact days (28.0 [IQR, 17.0-44.0] vs 29.0 [IQR, 18.0-45.0]), and lower continuity-of-care index (0.12 [IQR, 0.08-0.20] vs 0.13 [IQR, 0.08-0.21]) (Table 2). The MSSPs with the highest reported care management and coordination activities had a higher mean mortality rate than those in the bottom tertile (12.3% vs 11.9%).

In multivariate regression analyses, quality of care and health care utilization outcomes were measured per 100 beneficiaries for ease of presentation. We did not find statistically different prevention quality indicator admissions (−0.03; 95% CI, −0.91 to 0.85; *P* = .95) and 30-day readmissions (−0.02; 95% CI, −0.52 to 0.48; *P* = .94) between patients with complex needs attributed to MSSPs in the top care management and coordination index tertile and those attributed to MSSPs in the bottom tertile (Table 3). In patients in organizations with the highest reported care management and coordination activities, changes were not significant for E&M visits (−13.5; 95% CI, −116.3 to 89.4; *P* = .80), acute care or critical access hospital admissions (−0.3; 95% CI, −3.1 to 2.4; *P* = .81), inpatient days (−0.1; 95% CI, −1.0 to 0.9; *P* = .89), and number of ED visits (−0.2; 95% CI, −6.20 to 5.84; *P* = .95) compared with patients in MSSPs reporting less care management and coordination activities. We did not find statistically significant differences in spending (−$505; 95% CI, −$1756 to $746; *P* = .43 for change in total spending; −$192; 95% CI, −$731 to $347; *P* = .48 for change in post–acute care spending) for patients with complex needs in MSSPs in the top care management and coordination tertile compared with those in the bottom tertile. We estimated no difference in continuity-of-care scores (−0.004; 95% CI, −0.01 to 0.004; *P* = .33) or health care system contact days (−0.7; 95% CI, −2.4 to 1.0; *P* = .42) for patients attributed to MSSPs with the highest reported care management and coordination activities compared with those in MSSPs reporting less activity. Results were unchanged after restricting the sample to include beneficiaries with 3 or more chronic conditions and after adjusting for geography (eTable 6 in the Supplement).

**Table 3.** Association Between ACO Intensity of Care Management and Coordination Index and Outcomes for 1 402 582 Fee-for-Service Medicare Beneficiaries With Complex Needs, 2016^a^

Outcome Variable	Regression Model Coefficient (95% CI)^b^			
	Unadjusted		Covariate Adjusted^c^	
	ACO Care Management and Coordination Index in Second Tertile	ACO Care Management and Coordination Index in Third (Top) Tertile	ACO Care Management and Coordination Index in Second Tertile	ACO Care Management and Coordination Index in Third (Top) Tertile
Quality of care				
All-cause PQI admissions per 100 beneficiaries	−0.47 (−1.34 to 0.41)	0.20 (−1.48 to 1.88)	−0.31 (−1.19 to 0.58)	−0.03 (−0.91 to 0.85)
All-cause 30-d readmissions per 100 beneficiaries	−0.14 (−0.55 to 0.27)	0.12 (−0.78 to 1.02)	0.03 (−0.40 to 0.46)	−0.02 (−0.52 to 0.48)
Health care utilization				
E&M visits in ambulatory settings per 100 beneficiaries	−56.00 (−164.49 to 52.49)	−7.87 (−137.36 to 121.61)	−42.00 (−137.82 to 53.82)	−13.46 (−116.27 to 89.35)
Acute care or critical access hospital admissions per 100 beneficiaries	−1.23 (−4.01 to 1.55)	0.77 (−6.39 to 7.92)	−0.01 (−3.12 to 3.14)	−0.35 (−3.11 to 2.41)
Inpatient days	−0.75 (−2.03 to 0.53)	0.64 (−2.67 to 3.94)	−0.25 (−1.01 to 0.50)	−0.06 (−1.01 to 0.88)
ED visits per 100 beneficiaries	0.05 (−4.83 to 4.94)	0.89 (−8.40 to 10.18)	1.42 (−4.14 to 6.97)	−0.18 (−6.20 to 5.84)
Spending				
Total	−587 (−2076 to 901)	−225 (−2855 to 2406)	−101 (−1448 to 1246)	−505 (−1756 to 746)
PAC	−458 (−1112 to 196)	−64 (−1250 to 1121)	−254 (−762 to 254)	−192 (−731 to 347)
Interactions with the health care system				
Health care system contact days	−1.38 (−3.35 to 0.58)	−0.55 (−2.87 to 1.76)	−1.03 (−2.70 to 0.65)	−0.68 (−2.35 to 0.99)
Continuity-of-care index	−0.005 (−0.01 to 0.003)	−0.005 (−0.01 to 0.004)	−0.006 (−0.01 to 0.002)	−0.004 (−0.01 to 0.004)

Abbreviations: ACO, accountable care organization; ED, emergency department; E&M, evaluation and management; PAC, post–acute care; PQI, prevention quality indicator.

^a^ Includes beneficiaries 66 years or older.

^b^ Least squares regressions were estimated for all outcome variables, and regression coefficients with the associated 95% CIs are reported. Heteroscedasticity robust SEs (not reported) were clustered at the ACO level. Each model represents a single regression for each outcome variable, and all ACO care management and coordination index tertiles were jointly estimated.

^c^ Regressions were adjusted for cohort entry flags (frail and having multiple chronic conditions and an interaction term for being frail and having multiple chronic conditions), demographics (age, sex, race/ethnicity group, and high poverty status), dual eligibility for Medicaid status, and nursing home residency.

## Discussion

To measure whether ACO efforts to manage and coordinate care are associated with health care utilization and spending outcomes in groups likely to benefit from such services, we used data from the NSACO to form a care management and coordination index that plausibly targets older patients with complex health needs. We found wide variation in reported care management and coordination activities across MSSP participants. For example, when index scores were ranked by the level of care management activity reported, we observed large differences in the use of evidence-based approaches to care transitions for hospitalized patients. In multivariate analyses of patient-level measures for 244 ACO respondents, we found that high levels of care management and coordination intensity were not associated with significant differences in hospitalizations, inpatient days, E&M visits in ambulatory settings, spending, and health care system contact days despite large samples of beneficiaries in each group of ACOs.

Our results are in line with recent ACO literature^4,20^ noting the limitations of care management and coordination. This earlier work^4,20^ on ACOs used indirect assessment of care management and coordination activities. Our study is unique because we linked health care organization information on management and coordination activities with claims-based performance data, permitting study of whether and how measures of care use and quality are associated with an ACO’s care management and coordination efforts. Prior studies^4,20^ that combined many ACOs implementing limited management and coordination efforts with those intensively pursuing evidence-based strategies reported a lack of measured benefit even if the strategies used by some ACOs were effective. In articles^4,21^ that examined ACO performance compared with non-ACO performance in clinically vulnerable populations, some savings per beneficiary were found in the clinically vulnerable groups; however, how the ACOs achieved those savings was undefined.

The evidence base around care transition interventions, such as home visits after discharge, use of care navigators, and other features included in the care management and coordination index, is substantial, with randomized clinical trials^22,23,24,25^ supporting their efficacy at reducing hospitalizations and spending. Our work can be viewed as augmenting the evidence on how such strategies may play out in the community. When focusing on whether care management and coordination efforts by ACOs were associated with outcomes in older adults with complex needs, we did not find any statistically significant associations between care management and coordination and measures of care quality, utilization, spending, or interactions with the health care system. Findings in the care transition interventions literature suggest that a reduction in cost and utilization outcomes measured in our study may be desirable except for E&M visits and continuity of care, for which a favorable direction of the association was less clear.

We believe that the first step to better understanding the promise or limitations of care management and coordination is a better understanding of what health care organizations are implementing to address care needs for patients with complex needs. Given ample evidence to support the care management and coordination strategies that we queried^13,26,27,28,29,30,31,32^ and given external factors, such as the hospital readmission reduction program, which might add incentives to provider organizations to implement strategies for patients most at risk of hospitalization, we found a low reported use of evidence-based strategies designed to reduce readmission among ACOs in the bottom tertile of care management and coordination activities.

This study contributes to an important research gap—assessing the association between the self-reported care coordination activity of ACOs with performance for vulnerable Medicare beneficiaries. Mixed-methods work appears to be needed for care coordination activities to help better explain why evidence-based strategies do not generate the expected changes in spending, utilization, and quality.

Although our study is not definitive and includes several limitations, our estimates on various outcomes are indistinguishable from zero, indicating a call to invest in these areas with caution and to track local results in careful evaluations. Our results suggest that organizations should consider the effectiveness of investing heavily in care coordination activities that are difficult to implement. These results have the potential to inform practitioners and policy makers about ACO activities under the umbrella of care coordination to identify new areas of focus or improvement. As ACOs focus on expanding care coordination and management efforts, it is important to continually evaluate the effectiveness of those programs for different patient populations.

### Limitations

Our results should be interpreted considering several limitations. Measures of care management and coordination were self-reported by ACO leaders who may have incomplete information on care management activity at clinical front lines regarding the quantity and quality of services provided. We were unable to measure and control for variability in the case loads and for care management and coordination program delivery and duration at the ACO level.

Furthermore, there may be substantial differences in execution of reported activities that may result in important heterogeneity in the effectiveness of these activities. This is a challenge for any innovation being diffused to a heterogeneous set of practices with heterogeneous capabilities. In this article, we aimed to measure whether these efforts had an association. Although we did not find significant differences in cost or utilization outcomes and were unable to measure patient experience and physician satisfaction, care management and coordination programs might improve these outcomes.^33^ Although our study focused on the largest Medicare ACO program, the MSSP, our results may not be generalizable to other models.

The most important limitation of our study is its cross-sectional nature, which does not allow for causal interpretation. Because we used claims-based methods, residual, unmeasured differences between the comparison groups were possible. If ACOs with high levels of care management had sicker patients not captured by our claims-based measures, illness would have not been adequately adjusted for and an underlying association between care management and coordination and outcomes would not have been observed. Analyses using longitudinal data would allow the evaluation of activity duration and intensity and the assessment of the association of length of exposure to care management and coordination with outcomes. Although we used the most recent data available and closer to the time of our survey, we recognize that the claims predate our survey data. The lack of statistical differences in beneficiary outcomes across ACOs could be justified if ACOs had statistically indistinguishable levels of care management and coordination activities in 2016. However, it seems unlikely that ACOs with comprehensive care management and coordination activities began those efforts only after 2016. Another limitation is that we categorized MSSPs using an index of distinct care management and coordination activities that we hypothesized could influence care quality, outcomes, and costs for patients with complex needs, but the measure does not distinguish the individual components of care management activity. We did, however, present the underlying distribution of components contributing to the index.

Although our survey questions asked about the basic structural components that facilitate care management, they were not exhaustive. Additional components of care management and coordination occurring in conjunction with or outside the health care system (social and economic support, transportation, and food and housing)^6^ are important in caring for the whole patient across settings. Also, ACOs with high reported care management and coordination activities had fewer participants with nonwhite race/ethnicity compared with those reporting less activity and non–survey respondents, suggesting that the survey may tell us little about organizations that serve nonwhite populations. Although our study is not definitive, our estimates on various outcomes suggest the potential need for caution in future investments in care management and coordination.

## Conclusions

In this study, the ACO self-reports of care management and coordination capacity were not associated with differences in spending or measured outcomes for patients with complex needs. Future efforts to care for patients with complex needs should assess whether strategies found to be effective in other settings are being used, and if so, why they fail to meet expectations.

## Notes

Supplement. **eTable 1.** The NSACO Care Management and Coordination Index Score **eTable 2.** Claim-Based Frailty Indicators Adapted From Kim and Schneeweiss (2014) **eTable 3.** Definition of Chronic Conditions Based on 2016 Hierarchical Condition Categories (HCCs) (Categories Were Chosen for Chronic Nature and Association With Mortality and Costs) **eTable 4.** Description of Provider Number, Place of Service (POS) and Common Procedure Terminology (CPT) Codes Defining Nursing Facility Utilization Updated From Yun et al (2010) **eTable 5.** NSACO Respondents Versus Non-Respondent Analysis: Descriptive Characteristics of Fee-for-Service Medicare Beneficiaries With Complex Needs Attributed to ACO in 2016 **eTable 6.** Association Between ACO Intensity of Care Management and Coordination Index and Outcomes for Fee-for-Service Medicare Beneficiaries With Complex Needs, 2016
