# Supplementary material for: Association Between Care Management and Outcomes Among Patients With Complex Needs in Medicare Accountable Care Organizations
Source: JAMA Netw Open. 2019 Jul 12;2(7):e196939. doi: 10.1001/jamanetworkopen.2019.6939 (PMC6628588; doi:10.1001/jamanetworkopen.2019.6939)
Supplement: Supplement. — eTable 1. The NSACO Care Management and Coordination Index Score eTable 2. Claim-Based Frailty Indicators Adapted From Kim and Schneeweiss (2014) eTable 3. Definition of Chronic Conditions Based on 2016 Hierarchical Condition Categories (HCCs) (Categories Were Chosen for Chronic Nature and Association With Mortality and Costs) eTable 4. Description of Provider Number, Place of Service (POS) and Common Procedure Terminology (CPT) Codes Defining Nursing Facility Utilization Updated From Yun et al (2010) eTable 5. NSACO Respondents Versus Non-Respondent Analysis: Descriptive Characteristics of Fee-for-Service Medicare Beneficiaries With Complex Needs Attributed to ACO in 2016 eTable 6. Association Between ACO Intensity of Care Management and Coordination Index and Outcomes for Fee-for-Service Medicare Beneficiaries With Complex Needs, 2016 [file jamanetwopen-2-e196939-s001.pdf]

Supplementary Online Content

Ouayogodé MH, Mainor AJ, Meara E, Bynum JPW, Colla CH. Association between care management and outcomes among patients with complex needs in Medicare accountable care organizations. *JAMA Netw Open*. 2019;2(7):e196939. doi:10.1001/jamanetworkopen.2019.6939

eTable 1. The NSACO Care Management and Coordination Index Score

eTable 2. Claim-Based Frailty Indicators Adapted From Kim and Schneeweiss (2014)

eTable 3. Definition of Chronic Conditions Based on 2016 Hierarchical Condition Categories (HCCs) (Categories Were Chosen for Chronic Nature and Association With Mortality and Costs)

eTable 4. Description of Provider Number, Place of Service (POS) and Common Procedure Terminology (CPT) Codes Defining Nursing Facility Utilization Updated From Yun et al (2010)

eTable 5. NSACO Respondents Versus Non-Respondent Analysis: Descriptive Characteristics of Fee-for-Service Medicare Beneficiaries With Complex Needs Attributed to ACO in 2016

eTable 6. Association Between ACO Intensity of Care Management and Coordination Index and Outcomes for Fee-for-Service Medicare Beneficiaries With Complex Needs, 2016

This supplementary material has been provided by the authors to give readers additional information about their work.

© 2019 Ouayogodé MH et al. *JAMA Network Open.*

eTable 1. The NSACO Care Management/Coordination Index Score*

Question	Response Options	Points Value
Do you segment high-risk patients into sub-groups based on common needs (e.g., frailty, mental illness, similar combinations of chronic conditions)?	• No<br>• Yes	0<br>1
For patients attributed to the ACO, to what extent is a system in place for predictive risk stratification?	9 point Likert Scale<br>• 1-3: Little or no ability to identify and target patients<br>• 4-6: Some ability to identify and target patients<br>• 7-9: Comprehensive ability to identify and target patients	 0<br>1<br>2
To what extent are chronic care coordination processes and programs in place to manage patients with high-need, high-cost chronic illnesses?	9 point Likert Scale<br>• 1-3: Few or no chronic care coordination processes or programs in place<br>• 4-6: Some chronic care coordination processes or programs in place<br>• 7-9: Comprehensive chronic care coordination processes or programs in place	 0<br>1<br>2
To what extent are processes in place for clinicians to encourage ACO patients to be actively involved in decisions involving their care and self–management of their conditions?	9 point Likert Scale<br>• 1-3: Few or no processes in place<br>• 4-6: Some processes in place<br>• 7-9: Comprehensive program in place	 0<br>1<br>2
To what extent are systems in place to assure smooth transitions of care across all practice settings including hospitals, long–term care, home care, adult day care, and community–based health and social services as needed?	9 point Likert Scale<br>• 1-3: Few or no systems in place<br>• 4-6: Some systems in place<br>• 7-9: Nearly all/all necessary systems in place	 0<br>1<br>2
For how many of your ACO-attributed hospitalized patients undergoing a care transition to home or a post-acute care facility receive the following services to reduce the risk of readmission?		
a. Medication reconciliation	• Don't Know, None, Some<br>• Most, All	0<br>1
b. Telephone follow-up (within 72 hours of discharge)	• Don't Know, None, Some<br>• Most, All	0<br>1
c. In-home follow-up (within 72 hours of discharge)	• Don't Know, None, Some<br>• Most, All	0<br>1
d. Standardized process in place to ensure timely follow-up with primary or specialty care	• Don't Know, None, Some<br>• Most, All	0<br>1
e. Discharge summaries are transmitted to clinicians accepting care of the patient	• Don't Know, None, Some<br>• Most, All	0<br>1

© 2019 Ouayogodé MH et al. *JAMA Network Open.*

Question	Response Options	Points Value
Do you segment high-risk patients into sub-groups based on common needs (e.g., frailty, mental illness, similar combinations of chronic conditions)?	• No<br>• Yes	0<br>1
f. Use of a patient navigator or care manager while patient is in the hospital	• Don't Know, None, Some<br>• Most, All	0<br>1
g. Use of a care manager or health coach after discharge	• Don't Know, None, Some<br>• Most, All	0<br>1

Abbreviations: NSACO: national survey of accountable care organizations, ACO: accountable care organization

*We narrowed the index to incorporate 12 variables from six question stems. Each variable was assigned a point value of 0, 1, or 2 for a total of 0-16 points in the index. Our index is also highly correlated with an index based on a factor analysis (non-parametric ρ= 0.94).

Factor loadings (3 Factors retained) and unique variances based on a principal component analysis without rotation for 12 items from the NSACO Wave 4: N=406 NSACO Wave 4 respondents including ACOs with Medicare, Medicaid, and Commercial contracts

Variable	Factor1	Factor 2	Factor3	Uniquenes s
Reports segments high risk patients (1=Yes)	0.19	0.14	0.04	0.95
Predictive risk stratification (1-9 scale)	0.34	0.47	0.18	0.63
Chronic Care Management (processes in place 1-9 scale)	0.51	0.43	0.19	0.51
Shared Decision making (processes in place 1-9 scale)	0.42	0.42	0.09	0.64
Care transitions (processes in place 1-9 scale)	0.67	0.29	0.14	0.44
Medication reconciliation (how many receive) (1=Most or all patients)	0.58	-0.41	0.23	0.44
Use Patient Navigator or Care Manager (1=Most or all patients)	0.64	-0.04	-0.36	0.46
Standardized process in place to ensure timely follow up.. (1=Most or all patients)	0.84	-0.29	0.04	0.20
Discharge summaries transmitted to practitioners… (1=Most or all patients)	0.65	-0.35	0.17	0.43
Telephone Follow-up within 72 hours of discharge (1=Most or all patients)	0.79	-0.22	0.14	0.30
In home follow-up within 72 hours of discharge (1=Most or all patients)	0.56	0.11	-0.33	0.57
Use of care manager or health coach after discharge (1=Most or all patients)	0.82	0.08	-0.34	0.20

Abbreviations: NSACO: national survey of accountable care organizations, ACO: accountable care organization

eTable 2. Claim-based frailty indicators adapted from Kim and Schneeweiss (2014)*

**ICD 10's and HCPCS codes**	
Abnormality of gait	ICD-10: R26.0, R26.1, R26.89, R26.9
Malnutrition/ abnormal loss of weight and underweight	ICD-10: R63.4, R63.6
Adult failure to thrive	ICD-10: R62.7
Cachexia	ICD-10: R64
Debility	ICD-10: R53.81
Difficulty in walking	ICD-10: R26.2
Fall	ICD-10: Z91.81
Muscular wasting and disuse atrophy	ICD-10: M62.50
Muscle weakness	ICD-10: M62.81
Decubitus ulcer of skin/pressure ulcer	ICD-10: L89.004,L89.014,L89.024,L89.104,L89.114,L89.124,L89.134,L89.144,L89.154,L89.204,L89.214,L89.224,L89.304,L89.314,L89.324,L89.44,L89.504,L89.514,L89.524,L89.604,L89.614,L89.624,L89.814,L89.894,L89.94,L89.000,L89.003,L89.010,L89.013,L89.020,L89.023,L89.100,L89.103,L89.110,L89.113,L89.120,L89.123,L89.130,L89.133,L89.140,L89.143,L89.150,L89.153,L89.200,L89.203,L89.210,L89.213,L89.220,L89.223,L89.300,L89.303,L89.310,L89.313,L89.320,L89.323,L89.43,L89.45,L89.500,L89.503,L89.510,L89.513,L89.520,L89.523,L89.600,L89.603,L89.610,L89.613,L89.620,L89.623,L89.810,L89.813,L89.890,L89.893,L89.93,L89.95,L89.002,L89.012,L89.022,L89.102,L89.112,L89.122,L89.132,L89.142,L89.152,L89.202,L89.212,L89.222,L89.302,L89.312,L89.322,L89.42,L89.502,L89.512,L89.522,L89.602,L89.612,L89.622,L89.812,L89.892,L89.92,L89.000,L89.001,L89.002,L89.003,L89.004,L89.009,L89.010,L89.011,L89.012,L89.013,L89.014,L89.019,L89.020,L89.021,L89.022,L89.023,L89.024,L89.029,L89.100,L89.101,L89.102,L89.103,L89.104,L89.109,L89.110,L89.111,L89.112,L89.113,L89.114,L89.119,L89.120,L89.121,L89.122,L89.123,L89.124,L89.129,L89.130,L89.131,L89.132,L89.133,L89.134,L89.139,L89.140,L89.141,L89.142,L89.143,L89.144,L89.149,L89.150,L89.151,L89.152,L89.153,L89.154,L89.159,L89.200,L89.201,L89.202,L89.203,L89.204,L89.209,L89.210,L89.211,L89.212,L89.213,L89.214,L89.219,L89.220,L89.221,L89.222,L89.223,L89.224,L89.229,L89.300,L89.301,L89.302,L89.303,L89.304,L89.309,L89.310,L89.311,L89.312,L89.313,L89.314,L89.319,L89.320,L89.321,L89.322,L89.323,L89.324,L89.329,L89.40,L89.41,L89.42,L89.43,L89.44,L89.45,L89.500,L89.501,L89.502,L89.503,L89.504,L89.509,L89.510,L89.511,L89.512,L89.513,L89.514,L89.519,L89.520,L89.521,L89.522,L89.523,L89.524,L89.529,L89.600,L89.601,L89.602,L89.603,L89.604,L89.609,L89.610,L89.611,L89.612,L89.613,L89.614,L89.619,L89.620,L89.621,L89.622,L89.623,L89.624,L89.629,L89.810,L89.811,L89.812,L89.813,L89.814,L89.819,L89.890,L89.891,L89.892,L89.893,L89.894,L89.899,L89.90,L89.91,L89.92,L89.93,L89.94,L89.95
Senility without mention of psychosis	ICD-10: R41.81

© 2019 Ouayogodé MH et al. *JAMA Network Open.*

Durable Medical Equipment use (cane, walker, bath equipment, commode)	HCPCS: E0100, E0105, E0130, E0135, E0140, E0141, E0143, E0144, E0147-E0149, E0160-E0171 (BETOS CPT: D1E)
Malaise and fatigue	ICD-10: R53.2,R53.82,G93.3,R53.1,R53.81,R53.83
Nursing or personal care services	HCPCS: T1000-T1005, T1019-T1022, T1030, T1031 (BETOS CPT: Z2)

*We used *International Statistical Classification of Diseases and Related Health Problems, Tenth Revision* (ICD-10) and Healthcare Common Procedure Coding System (HCPCS) codes to identify beneficiaries in the frail elderly cohort. Beneficiaries were included in the frail elderly cohort if they had at least two frailty indicators including: abnormality of gait, malnutrition/abnormal loss of weight and underweight, adult failure to thrive, cachexia, debility, difficulty in walking, fall, muscular wasting and disuse atrophy, muscle weakness, decubitus ulcer of skin/pressure ulcer, senility without mention of psychosis, durable medical equipment use, malaise and fatigue, nursing or personal care services).

eTable 3. Definition of chronic conditions based on 2016 Hierarchical Condition Categories (HCC) (categories were chosen for chronic nature and association with mortality and costs)

**ICD-10's for Each HCC - Label and Number**	
**Coronary artery disease (CAD)**	**HCC86:** I21.01, I21.02, I21.09, I21.11, I21.19, I21.21,I21.29, I21.3, I21.4, I21.9, I21.A1, I21.A9, I12.9, I22.0, I22.1, I22.2, I22.8, I22.9, I23.4, I23.5, I51.1, I51.2
	**HCC87:** I20.0, I23.0,123.1,123.2,I23.3, I23.6,123.7,I23.8, I24.0,I24.1, I24.8, I24.9,I25.110, I25.700, I25.710, I25.720, I25.730, I25.750,I25.760, I25.790
	**HCC88:** I20.1,I20.8,I20.9,I25.111, I25.118,I25.119, I25.701,I25.708,I25.709,I25.711,I25.718,I25.719, I25.721,I25.728,I25.729,I25.731,I25.738,I25.739,I25.751,I25.758,I25.759,I25.761,I25.768,I25.769,I25.791,I25.798,I25.799
**Cerebral hemorrhage/stroke**	**HCC99:** I60.00, I60.01, I60.02, I60.10, I60.11,I60.12,I60.20,I60.21,I60.22, I60.30, I60.31,I60.32,I60.4,I60.50,I60.51,I60.52,I60.6,I60.7,I60.8,I60.9,I61.0,I61.1,I61.2,I61.3,I61.4,I61.5,I61.6,I61.8,I61.9,I62.00,I62.01,I62.02,I62.03,I62.1,I62.9
	**HCC100:** I63.00,I63.011,I63.012,I63.019,I63.02,I63.031,I63.032,I63.039,I63.09,I63.10,I63.111,I63.112,I63.119,I63.12,I63.131,I63.132,I63.139,I63.19,I63.20,I63.211,I63.212,I63.219,I63.22,I63.231,I63.232,I63.239,I63.29,I63.30,I63.311,I63.312,I63.319,I63.321,I63.322,I63.329,I63.331,I63.332,I63.339,I63.341,I63.342,I63.349,I63.39,I63.40,I63.411,I63.412,I63.419,I63.421,I63.421,I63.422,I63.429,I63.431,I63.432,I63.439,I63.441,I63.442,I63.449,I63.49,I63.50,I63.511,I63.512,I63.519,I63.521,I63.522,I63.529,I63.531,I63.532,I63.539,I63.541,I63.542,I63.549,I63.59,I63.6,I63.8,I63.9,I97.810,I97.811,I97.820,I97.821
	**HCC103:** G81.10,G81.11,G81.12,G81.13,G81.14,G81.00,G81.01,G81.02,G81.03,G81.04,G81.90,G81.91, G81.92,G81.93,G81.94,I69.051,I69.052,I69.053,I69.054,I69.059,I69.151,I69.152,I69.153,I69.154,I69.159,I69.251,I69.252,I69.253,I69.254,I69.259,I69.351,I69.352,I69.353,I69.354,I69.359,I69.851,I69.852,I69.853,I69.854,I69.859,I69.951,I69.952,I69.953,I69.954,I69.959
**Cancer**	**HCC8:** C77.1,C77.2,C77.4,C77.5,C77.8,C78.00,C78.01,C78.02,C78.1,C78.2,C78.30,C78.39,C78.4,C78.5, C78.6,C78.7,C78.80,C78.89,C79.00,C79.01,C79.02,C79.10,C79.11,C79.19,C79.2,C79.31,C79.32, C79.40,C79.49,C79.51,C79.52,C79.60,C79.61,C79.62,C79.70,C79.71,C79.72,C79.89,C79.9,C7B.00,C7B.01,C7B.02,C7B.03,C7B.04,C7B.09,C7B.1,C7B.8,C80.0,C91.00,C91.01,C91.02,C92.00,C92.02,C92.40,C92.41,C92.42,C92.50,C92.51,C92.52,C92.60,C92.61,C92.62,C92.90,C92.91,C92.92, C92.A0,C92.A1,C92.A2,C93.00,C93.01,C93.02,C94.00,C94.01,C94.02,C94.20,C94.21,C94.22,C94.40,C94.41,C94.42, C95.00,C95.01,C95.02
	**HCC9:** C15.3,C15.4,C15.5,C15.8,C15.9,C16.0,C16.1,C16.2,C16.3,C16.4,C16.5,C16.6,C16.8,C16.9,C17.0, C17.1,C17.2,C17.3,C17.8,C17.9,C22.0,C22.1,C22.2,C22.3,C22.4,C22.7,C22.8,C22.9,C23,C24.0,C24.1,C24.8,C24.9,C25.0,C25.1,C25.2,C25.3,C25.4,C25.7,C25.8,C25.9,C33,C34.00,C34.01,C34.02,C34.10,C34.11,C34.12,C34.2,C34.30,C34.31,C34.32,C34.80,C34.81,C34.82,C34.90,C34.91,C34.92,C38.4,C45.0,C45.1,C45.2,C45.7,C45.9,C48.0,C48.1,C48.2,C48.8,C90.00,C90.01,C90.02,C90.10,C90.11,C90.12,C90.20,C90.21,C90.22,C92.10,C92.11,C92.12,C92.20,C92.21,C92.22,C92.30,C92.31,C92.32, C92.Z0,C92.Z1,C92.Z2,C92.90,C92.91,C92.92,C93.10,C93.11,C93.12,C93.30,C93.31,C93.32,C93.90,C93.91,C93.92,C93.Z0,C93.Z1,C93.Z2,C94.30,C94.31,C94.32,C94.80,C94.81,C94.82

© 2019 Ouayogodé MH et al. *JAMA Network Open.*

	**HCC10:** C40.00,C40.01,C40.02,C40.10,C40.11,C40.12,C40.20,C40.21,C40.22,C40.30,C40.31,C40.32,C40.80,C40.81,C40.82,C40.90,C40.91,C40.92,C41.0,C41.1,C41.2,C41.3,C41.4,C41.9,C46.0,C46.1,C46.2,C46.3,C46.4,C46.50,C46.51,C46.52,C46.7,C46.9,C47.0,C47.10,C47.11,C47.12,C47.20,C47.21,C47.22,C47.3,C47.4,C47.5,C47.6,C47.8,C47.9,C49.0,C49.10,C49.11,C49.12,C49.20,C49.21,C49.22,C49.3,C49.4,C49.5,C49.6,C49.8,C49.9,C56.1,C56.2,C56.9,C57.00,C57.01,C57.02,C57.10,C57.11,C57.12,C57.20,C57.21,C57.22,C57.3,C57.4,C58,C70.00,C70.01,C70.09,C71.0,C71.1,C71.2,C71.3,C71.4,C71.5,C71.6,C71.7,C71.8,C71.9,C72.0,C72.1,C72.20,C72.21,C72.22,C72.30,C72.31,C72.32,C72.40,C72.41,C72.42,C72.50,C72.59,C72.9,C74.00,C74.01,C74.02,C74.10,C74.11,C74.12,C74.90,C74.91,C74.92,C75.1,C75.2,C75.3,C77.3,C77.9,C79.2,C79.81,C79.82,C81.00,C81.01,C81.02,C81.03,C81.04,C81.05,C81.06,C81.07,C81.08,C81.09,C81.10,C81.11,C81.12,C81.13,C81.14,C81.15,C81.16,C81.17,C81.18,C81.19,C81.20,C81.21,C81.22,C81.23,C81.24,C81.25,C81.26,C81.27,C81.28,C81.29,C81.30,C81.31,C81.32,C81.33,C81.34,C81.35,C81.36,C81.37,C81.38,C81.39,C81.40,C81.41,C81.42,C81.43,C81.44,C81.45,C81.46,C81.47,C81.48,C81.49,C81.70,C81.71,C81.72,C81.73,C81.74,C81.75,C81.76,C81.77,C81.78,C81.79,C81.90,C81.91,C81.92,C81.93,C81.94,C81.95,C81.96,C81.97,C81.98,C81.99,,C82.00,C82.01,C82.02,C82.03,C82.04,C82.05,C82.06,C82.07,C82.08,C82.09,C82.10,C82.11,C82.12,C82.13,C82.14,C82.15,C82.16,C82.17,C82.18,C82.19,C82.20,C82.21,C82.22,C82.23,C82.24,C82.25,C82.26,C82.27,C82.28,C82.29,C82.30,C82.31,C82.32,C82.33,C82.34,C82.35,C82.36,C82.37,C82.38,C82.39,C82.40,C82.41,C82.42,C82.43,C82.44,C82.45,C82.46,C82.47,C82.48,C82.49,C82.50,C82.51,C82.52,C82.53,C82.54,C82.55,C82.56,C82.57,C82.58,C82.59,C82.60,C82.61,C82.62,C82.63,C82.64,C82.65,C82.66,C82.67,C82.68,C82.69,C82.80,C82.81,C82.82,C82.83,C82.84,C82.85,C82.86,C82.87,C82.88,C82.89,C82.90,C82.91,C82.92,C82.93,C82.94,C82.95,C82.96,C82.97,C82.98,C82.99,C83.00,C83.01,C83.02,C83.03,C83.04,C83.05,C83.06,C83.07,C83.08,C83.09,C83.10,C83.11,C83.12,C83.13,C83.14,C83.15,C83.16,C83.17,C83.18,C83.19,C83.30,C83.31,C83.32,C83.33,C83.34,C83.35,C83.36,C83.37,C83.38,C83.39,C83.50,C83.51,C83.52,C83.53,C83.54,C83.55,C83.56,C83.57,C83.58,C83.59,C83.70,C83.71,C83.72,C83.73,C83.74,C83.75,C83.76,C83.77,C83.78,C83.79,C83.80,C83.81,C83.82,C83.83,C83.84,C83.85,C83.86,C83.87,C83.88,C83.89,C83.90,C83.91,C83.92,C83.93,C83.94,C83.95,C83.96,C83.97,C83.98,C83.99,C84.00,C84.01,C84.02,C84.03,C84.04,C84.05,C84.06,C84.07,C84.08,C84.09,C84.10,C84.11,C84.12,C84.13,C84.14,C84.15,C84.16,C84.17,C84.18,C84.19,C84.40,C84.41,C84.42,C84.43,C84.44,C84.45,C84.46,C84.47,C84.48,C84.49,C84.60,C84.61,C84.62,C84.63,C84.64,C84.65,C84.66,C84.67,C84.68,C84.69,C84.70,C84.71,C84.72,C84.73,C84.74,C84.75,C84.76,C84.77,C84.78,C84.79,C84.90,C84.91,C84.92,C84.93,C84.94,C84.95,C84.96,C84.97,C84.98,C84.99,C84.A0,C84.A1,C84.A2,C84.A3,C84.A4,C84.A5,C84.A6,C84.A7,C84.A8,C84.A9,C84.Z0,C84.Z1,C84.Z2,C84.Z3,C84.Z4,C84.Z5,C84.Z6,C84.Z7,C84.Z8,C84.Z9,C85.10,C85.11,C85.12,C85.13,C85.14,C85.15,C85.16,C85.17,C85.18,C85.19,C85.20,C85.21,C85.22,C85.23,C85.24,C85.25,C85.26,C85.27,C85.28,C85.29,C85.80,C85.81,C85.82,C85.83,C85.84,C85.85,C85.86,C85.87,C85.88,C85.89,C85.90,C85.91,C85.92,C85.93,C85.94,C85.95,C85.96,C85.97,C85.98,C85.99,C86.0,C86.1,C86.2,C86.3,C86.4,C86.5,C86.6,C88.2,C88.3,C88.4,C88.8,C88.9,C90.30,C90.31,C90.32,C91.10,C91.11,C91.12,C91.30,C91.31,C91.32,C91.40,C91.41,C91.42,C91.50,C91.51,C91.52,C91.60,C91.61,C91.62,C91.90,C91.91,C91.92,C91.A0,C91.A1,C91.A2,C91.Z0,C91.Z1,C91.Z2,C95.10,C95.11,C95.12,C95.90,C95.91,C95.92,C96.0,C96.2,C96.4,C96.5,C96.6,C96.9,C96.A,C96.Z
	**HCC11:** ,C01,C02.0,C02.1,C02.2,C02.3,C02.4,C02.8,C02.9,C03.0,C03.1,C03.9,C04.0,C04.1,C04.8,C04.9,C05.0,C05.1,C05.2,C05.8,C05.9,C06.0,C06.1,C06.2,C06.80,C06.89,C06.9,C07,C08.0,C08.1,C08.9,C09.0,C09.1,C09.8,C09.9,C10.0,C10.1,C10.2,C10.3,C10.4,C10.8,C10.9,C11.0,C11.1,C11.2,C11.3,C11.8,C11.9,C12,C13.0,C13.1,C13.2,C13.8,C13.9,C14.0,C14.2,C14.8,C18.0,C18.1,C18.2,C18.3,C18.4,C18.5,C18.6,C18.7,C18.8,C18.9,C19,C20,C21.0,C21.1,C21.2,C21.8,C26.0,C26.1,C26.9,C30.0,C30.1,C31.0,C31.1,C31.2,C31.3,C31.8,C31.9,C32.0,C32.1,C32.2,C32.3,C32.8,C32.9,C37,C38.0,C38.1,C38.2,C38.3,C38.8,C39.0,C39.9,C51.0,C51.1,C51.2,C51.8,C51.9,C52,C53.0,C53.1,C53.8,C53.9,C57.7,C57.8,C57.9,C64.1,C64.2,C64.9,C65.1,C65.2,C65.9,C66.1,C66.2,C66.9,C67.0,C67.1,C67.2,C67.3,C67.4,C67.5,C67.6,C67.7,C67.8,C67.9,C68.0,C68.1,C68.8,C68.9

© 2019 Ouayogodé MH et al. *JAMA Network Open.*

	**HCC12:** C43.0,C43.10,C43.11,C43.12,C43.20,C43.21,C43.22,C43.30,C43.31,C43.39,C43.4,C43.51,C43.52,C43.59,C43.60,C43.61,C43.62,C43.70,C43.71,C43.72,C43.8,C43.9,C4A.0,C4A.10,C4A.11,C4A.12,C4A.20,C4A.21,C4A.22,C4A.30,C4A.31,C4A.39,C4A.4,C4A.51,C4A.52,C4A.59,C4A.60,C4A.61,C4A.62,C4A.70,C4A.71,C4A.72,C4A.8,C4A.9,C50.011,C50.012,C50.019,C50.021,C50.022,C50.029,C50.111,C50.112,C50.119,C50.121,C50.122,C50.129,C50.211,C50.212,C50.219,C50.221,C50.222,C50.229,C50.311,C50.312,C50.319,C50.321,C50.322,C50.329,C50.411,C50.412,C50.419,C50.421,C50.422,C50.429,C50.511,C50.512,C50.519,C50.521,C50.522,C50.529,C50.611,C50.612,C50.619,C50.621,C50.622,C50.629,C50.811,C50.812,C50.819,C50.821,C50.822,C50.829,C50.911,C50.912,C50.919,C50.921,C50.922,C50.929,C54.0,C54.1,C54.2,C54.3,C54.8,C54.9,C55,C60.0,C60.1,C60.2,C60.8,C60.9,C61,C62.00,C62.01,C62.02,C62.10,C62.11,C62.12,C62.90,C62.91,C62.92,C63.00,C63.01,C63.02,C63.10,C63.11,C63.12,C63.2,C63.7,C63.8,C63.9,C69.00,C69.01,C69.02,C69.10,C69.11,C69.12,C69.20,C69.21,C69.22,C69.30,C69.31,C69.32,C69.40,C69.41,C69.42,C69.50,C69.51,C69.52,C69.60,C69.61,C69.62,C69.80,C69.81,C69.82,C69.90,C69.91,C69.92,C73,C75.0,C75.4,C75.5,C75.8,C75.9,C76.0,C76.1,C76.2,C76.3,C76.40,C76.41,C76.42,C76.50,C76.51,C76.52,C76.8,C7A.00,C7A.010,C7A.011,C7A.012,C7A.019,C7A.020,C7A.021,C7A.022,C7A.023,C7A.024,C7A.025,C7A.026,C7A.029,C7A.090,C7A.091,C7A.092,C7A.093,C7A.094,C7A.095,C7A.096,C7A.098,C7A.1,C7A.8,C80.1,C80.2,D03.0,D03.10,D03.11,D03.12,D03.20,D03.21,D03.22,D03.30,D03.39,D03.4,D03.51,D03.52,D03.59,D03.60,D03.61,D03.62,D03.70,D03.71,D03.72,D03.8,D03.9,D18.02,D32.0,D32.1,D32.9,D33.0,D33.1,D33.2,D33.3,D33.4,D33.7,D33.9,D35.2,D35.3,D35.4,D42.0,D42.1,D42.9,D43.0,D43.1,D43.2,D43.3,D43.4,D43.8,D43.9,D44.3,D44.4,D44.5,D44.6,D44.7,D49.6E34.0,Q85.00,Q85.01,Q85.02,Q85.03,Q85.09,Q85.1,Q85.8,Q85.9
**Congestive heart failure (CHF)**	**HCC85:** A36.81,B33.24,I09.81,I11.0,I13.0,I13.2,I26.01,I26.02,I26.09,I27.0,I27.1,I27.2,I27.81,I27.89,I27.9,I28.0,I28.1,I28.8,I28.9,I42.0,I42.1,I42.2,I42.3,I42.4,I42.5,I42.6,I42.7,I42.8,I42.9,I43,I50.1,I50.20,I50.21,I50.22,I50.23,I50.30,I50.31,I50.32,I50.33,I50.40,I50.41,I50.42,I50.43,I50.9,I51.4,I51.5
**Connective tissue disorders**	**HCC40:** L40.50,L40.51,L40.52,L40.53,L40.54,L40.59,M02.30,M02.311,M02.312,M02.319,M02.321,M02.322,M02.329,M02.331,M02.332,M02.339,M02.341,M02.342,M02.349,M02.351,M02.352,M02.359,M02.361,M02.362,M02.369,M02.371,M02.372,M02.379,M02.38,M02.39,M06.00,M06.011,M06.012,M06.019,M06.021,M06.022,M06.029,M06.031,M06.032,M06.039,M06.041,M06.042,M06.049,M06.051,M06.052,M06.059,M06.061,M06.062,M06.069,M06.071,M06.072,M06.079,M06.09,M06.10,M06.111,M06.112,M06.119,M06.121,M06.122,M06.129,M06.131,M06.132,M06.139,M06.141,M06.142,M06.149,M06.151,M06.152,M06.159,M06.161,M06.162,M06.169,M06.171,M06.172,M06.179,M06.19,M06.20,M06.211,M06.212,M06.219,M06.221,M06.222,M06.229,M06.231,M06.232,M06.239,M06.241,M06.242,M06.249,M06.251,M06.252,M06.259,M06.261,M06.262,M06.269,M06.271,M06.272,M06.279,M06.29,M06.30,M06.311,M06.312,M06.319,M06.321,M06.322,M06.329,M06.331,M06.332,M06.339,M06.341,M06.342,M06.349,M06.351,M06.352,M06.359,M06.361,M06.362,M06.369,M06.371,M06.372,M06.379,M06.39,M06.40,M06.411,M06.412,M06.419,M06.421,M06.422,M06.429,M06.431,M06.432,M06.439,M06.441,M06.442,M06.449,M06.451,M06.452,M06.459,M06.461,M06.462,M06.469,M06.471,M06.472,M06.479,M06.49,M06.50,M06.511,M06.512,M06.519,M06.521,M06.522,M06.529,M06.531,M06.532,M06.539,M06.541,M06.542,M06.549,M06.551,M06.552,M06.559,M06.561,M06.562,M06.569,M06.571,M06.572,M06.579,M06.59,M06.60,M06.611,M06.612,M06.619,M06.621,M06.622,M06.629,M06.631,M06.632,M06.639,M06.641,M06.642,M06.649,M06.651,M06.652,M06.659,M06.661,M06.662,M06.669,M06.671,M06.672,M06.679,M06.69,M06.70,M06.711,M06.712,M06.719,M06.721,M06.722,M06.729,M06.731,M06.732,M06.739,M06.741,M06.742,M06.749,M06.751,M06.752,M06.759,M06.761,M06.762,M06.769,M06.771,M06.772,M06.779,M06.79,M06.80,M06.811,M06.812,M06.819,M06.821,M06.822,M06.829,M06.831,M06.832,M06.839,M06.841,M06.842,M06.849,M06.851,M06.852,M06.859,M06.861,M06.862,M06.869,M06.871,M06.872,M06.879,M06.89,M06.9,M08.00,M08.011,M08.012,M08.019,M08.021,M08.022,M08.029,M08.031,M08.032,M08.039,M08.041,M08.042,M08.04

© 2019 Ouayogodé MH et al. *JAMA Network Open.*

	9,M08.051,M08.052,M08.059,M08.061,M08.062,M08.069,M08.071,M08.072,M08.079,M08.08,M08.09,M08.1,M08.20,M08.211,M08.212,M08.219,M08.221,M08.222,M08.229,M08.231,M08.232,M08.239,M08.241,M08.242,M08.249,M08.251,M08.252,M08.259,M08.261,M08.262,M08.269,M08.271,M08.272,M08.279,M08.28,M08.29,M08.3,M08.40,M08.411,M08.412,M08.419,M08.421,M08.422,M08.429,M08.431,M08.432,M08.439,M08.441,M08.442,M08.449,M08.451,M08.452,M08.459,M08.461,M08.462,M08.469,M08.471,M08.472,M08.479,M08.48,M08.80,M08.811,M08.812,M08.819,M08.821,M08.822,M08.829,M08.831,M08.832,M08.839,M08.841,M08.842,M08.849,M08.851,M08.852,M08.859,M08.861,M08.862,M08.869,M08.871,M08.872,M08.879,M08.88,M08.89,M08.90,M08.911,M08.912,M08.919,M08.921,M08.922,M08.929,M08.931,M08.932,M08.939,M08.941,M08.942,M08.949,M08.951,M08.952,M08.959,M08.961,M08.962,M08.969,M08.971,M08.972,M08.979,M08.98,M08.99,M12.00,M12.011,M12.012,M12.019,M12.021,M12.022,M12.029,M12.031,M12.032,M12.039,M12.041,M12.042,M12.049,M12.051,M12.052,M12.059,M12.061,M12.062,M12.069,M12.071,M12.072,M12.079,M12.08,M12.09,M30.0,M30.1,M30.2,M30.3,M30.8,M31.0,M31.1,M31.2,M31.30,M31.31,M31.4,M31.5,M31.6,M31.7,M32.0,M32.10,M32.11,M32.12,M32.13,M32.14,M32.15,M32.19,M32.8,M32.9,M33.00,M33.01,M33.02,M33.09,M33.10,M33.11,M33.12,M33.19,M33.20,M33.21,M33.22,M33.29,M33.90,M33.91,M33.92,M33.99,M34.0,M34.1,M34.2,M34.81,M34.82,M34.83,M34.89,M34.9,M35.00,M35.01,M35.02,M35.03,M35.04,M35.09,M35.1,M35.2,M35.3,M35.5,M35.8,M35.9,M36.0,M36.8,M45.0,M45.1,M45.2,M45.3,M45.4,M45.5,M45.6,M45.7,M45.8,M45.9,M46.00,M46.01,M46.02,M46.03,M46.04,M46.05,M46.06,M46.07,M46.08,M46.09,M46.1,M46.50,M46.51,M46.52,M46.53,M46.54,M46.55,M46.56,M46.57,M46.58,M46.59,M46.80,M46.81,M46.82,M46.83,M46.84,M46.85,M46.86,M46.87,M46.88,M46.89,M46.90,M46.91,M46.92,M46.93,M46.94,M46.95,M46.96,M46.97,M46.98,M46.99,M48.8X1,M48.8X2,M48.8X3,M48.8X4,M48.8X5,M48.8X6,M48.8X7,M48.8X8,M48.8X9,M49.80,M49.81,M49.82,M49.83,M49.84,M49.85,M49.86,M49.87,M49.88,M49.89
**Chronic obstructive pulmonary disease (COPD)**	**HCC111:** J41.0,J41.1,J41.8,J42,J43.0,J43.1,J43.2,J43.8,J43.9,J44.0,J44.1,J44.9,J98.2,J98.3
**Diabetes**	**HCC17:** E08.00,E08.01,E08.10,E08.11,E08.641,E09.00,E09.01,E09.10,E09.11,E09.641,E10.10,E10.11,E10.641,E11.00,E11.01,E11.641,E13.00,E13.01,E13.10,E13.11,E13.641
	**HCC18:** E08.21,E08.22,E08.29,E08.311,E08.319,E08.321,E08.329,E08.331,E08.339,E08.341,E08.349,E08.351,E08.359,E08.36,E08.39,E08.40,E08.41,E08.42,E08.43,E08.44,E08.49,E08.51,E08.52,E08.59,E08.610,E08.618,E08.620,E08.621,E08.622,E08.628,E08.630,E08.638,E08.649,E08.65,E08.69,E08.8,E09.21,E09.22,E09.29,E09.311,E09.319,E09.321,E09.329,E09.331,E09.339,E09.341,E09.349,E09.351,E09.359,E09.36,E09.39,E09.40,E09.41,E09.42,E09.43,E09.44,E09.49,E09.51,E09.52,E09.59,E09.610,E09.618,E09.620,E09.621,E09.622,E09.628,E09.630,E09.638,E09.649,E09.65,E09.69,E09.8,E10.21,E10.22,E10.29,E10.311,E10.319,E10.321,E10.329,E10.331,E10.339,E10.341,E10.349,E10.351,E10.359,E10.36,E10.39,E10.40,E10.41,E10.42,E10.43,E10.44,E10.49,E10.51,E10.52,E10.59,E10.610,E10.618,E10.620,E10.621,E10.622,E10.628,E10.630,E10.638,E10.649,E10.65,E10.69,E10.8,E11.21,E11.22,E11.29,E11.311,E11.319,E11.321,E11.329,E11.331,E11.339,E11.341,E11.349,E11.351,E11.359,E11.36,E11.39,E11.40,E11.41,E11.42,E11.43,E11.44,E11.49,E11.51,E11.52,E11.59,E11.610,E11.618,E11.620,E11.621,E11.622,E11.628,E11.630,E11.638,E11.649,E11.65,E11.69,E11.8,E13.21,E13.22,E13.29,E13.311,E13.319,E13.321,E13.329,E13.331,E13.339,E13.341,E13.349,E13.351,E13.359,E13.36,E13.39,E13.40,E13.41,E13.42,E13.43,E13.44,E13.49,E13.51,E13.52,E13.59,E13.610,E13.618,E13.620,E13.621,E13.622,E13.628,E13.630,E13.638,E13.649,E13.65,E13.69,E13.8
	**HCC19:** E08.9,E09.9,E10.9,E11.9,E13.9,Z79.4

© 2019 Ouayogodé MH et al. *JAMA Network Open.*

	**HCC122:** E08.351,E08.359,E09.351,E09.359,E10.351,E10.359,E11.351,E11.359,E13.351,E13.359,H43.10,H43.11,H43.12,H43.13
Dementia	**RxHCC111:** G30.0,G30.1,G30.8,G30.9
	**RxHCC112:** A81.00,A81.01,A81.09,A81.1,A81.2,A81.81,A81.82,A81.83,A81.89,A81.9,E75.00,E75.01,E75.02,E75.09,E75.10,E75.11,E75.19,E75.23,E75.25,E75.29,E75.4,F01.50,F01.51,F02.80,F02.81,F03.90,F03.91,F06.0,F84.2,G31.01,G31.09,G31.1,G31.2,G31.81,G31.82,G31.83,G31.85,G31.89,G31.9,G93.7,G93.89,G93.9,I67.3
Hematologic/thrombotic disease	**HCC46:** D46.C,D57.00,D57.01,D57.02,D57.1,D46.0,D46.21,D46.22,D46.4,D46.9,D46.A,D46.B,D46.Z,D47.4,D57.20,D57.211,D57.212,D57.219,D57.40,D57.411,D57.412,D57.419,D57.80,D57.811,D57.812,D57.819,D59.0,D59.1,D59.2,D59.3,D59.4,D59.5,D59.6,D59.8,D59.9,D60.0,D60.1,D60.8,D60.9,D61.01,D61.09,D61.1,D61.2,D61.3,D61.82,D61.89,D61.9,D66,D67,D75.81
	**RxHCC215:** I70.208,I70.209,I70.211,I70.212,I70.213,I70.218,I70.219,I70.221,I70.222,I70.223,I70.228,I70.229,I70.291,I70.292,I70.293,I70.298,I70.299,I70.301,I70.302,I70.303,I70.308,I70.309,I70.311,I70.312,I70.313,I70.318,I70.319,I70.321,I70.322,I70.323,I70.328,I70.329,I70.391,I70.392,I70.393,I70.398,I70.399,I70.401,I70.402,I70.403,I70.408,I70.409,I70.411,I70.412,I70.413,I70.418,I70.419,I70.421,I70.422,I70.423,I70.428,I70.429,I70.491,I70.492,I70.493,I70.498,I70.499,I70.501,I70.502,I70.503,I70.508,I70.509,I70.511,I70.512,I70.513,I70.518,I70.519,I70.521,I70.522,I70.523,I70.528,I70.529,I70.591,I70.592,I70.593,I70.598,I70.599,I70.601,I70.602,I70.603,I70.608,I70.609,I70.611,I70.612,I70.613,I70.618,I70.619,I70.621,I70.622,I70.623,I70.628,I70.629,I70.691,I70.692,I70.693,I70.698,I70.699,I70.701,I70.702,I70.703,I70.708,I70.709,I70.711,I70.712,I70.713,I70.718,I70.719,I70.721,I70.722,I70.723,I70.728,I70.729,I70.791,I70.792,I70.793,I70.798,I70.799,I70.92,I71.2,I71.4,I71.6,I71.9,I72.0,I72.1,I73.1,I73.81,I73.89,I73.9,I79.1,I79.8,E08.52,E09.52,E10.52,E11.52,E13.52
HIV/AIDS	**HCC1:** B20, B97.35, Z21
Immune disease	**HCC47:** D61.810,D61.811,D61.818,D70.0,D70.1,D70.2,D70.3,D70.4,D70.8,D70.9,D71.,D72.0,D76.1,D76.2,D76.3,D76.3,D89.810,D89.811,D80.0,D80.1,D80.2,D80.3,D80.4,D80.5,D80.6,D80.7,D80.8,D80.9,D81.0,D81.1,D81.2,D81.3,D81.4,D81.5,D81.6,D81.7,D81.89,D81.9,D82.0,D82.1,D82.2,D82.3,D82.4,D82.8,D82.9,D83.0,D83.1,D83.2,D83.8,D83.9,D84.0,D84.8,D84.9,D89.3,D89.812,D89.813,D89.82,D89.89,D89.9
Liver disease	**HCC27:** I85.00,I85.01,I85.10,I85.11,K70.41,K71.11,K72.01,K72.10,K72.11,K72.90,K72.91,K76.6,K76.7,K76.81
	**HCC28:** K70.30,K70.31,K70.40,K70.41,K70.9,K74.3,K74.4,K74.5,K74.60,K74.69
	**HCC29:** B18.0,B18.1,B18.2,B18.8,B18.9,K73.0,K73.1,K73.2,K73.8,K73.9,K75.4
Parkinson's/Huntington's	**HCC78:** G10,G20,G21.11,G21.19,G21.2,G21.3,G21.4,G21.8,G21.9,G23.0,G23.1,G23.2,G23.8,G23.9,G9.03
Paralysis	**HCC70:** G82.50,G82.51,G82.52,G82.53,G82.54,R53.2,S14.111A,S14.111D,S14.111S,S14.112A,S14.112D,S14.112S,S14.113A,S14.113D,S14.113S,S14.114A,S14.114D,S14.114S,S14.115A,S14.115D,S14.115S,S14.116A,S14.116D,S14.116S,S14.117A,S14.117D,S14.117S,S14.118A,S14.118D,S14.118S,S14.119A,S14.119D,S14.119S
	**HCC71:** G82.20,G82.21,G82.22,S24.111A,S24.111D,S24.111S,S24.112A,S24.112D,S24.112S,S24.113A,S24.113D,S24.113S,S24.114A,S24.114D,S24.114S,S24.119A,S24.119D,S24.119S

© 2019 Ouayogodé MH et al. *JAMA Network Open.*

	**HCC72:** B00.82,B01.12,B02.24,G04.1,G04.89,G04.91,G05.4,G12.0,G12.1,G12.8,G12.9,G32.0,G37.3,G37.4,G834,G901,G95.0,G95.11,G95.19,G95.20,G95.29,G95.81,G95.89,G95.9,G992,Q00.0,Q00.1,Q00.2,Q01.0,Q01.1,Q01.2,Q01.8,Q01.9,Q02,Q03.0,Q03.1,Q03.8,Q03.9,Q04.0,Q04.1,Q04.2,Q04.3,Q04.4,Q04.5,Q04.6,Q04.8,Q04.9,Q05.0,Q05.1,Q05.2,Q05.3,Q05.4,Q05.5,Q05.6,Q05.7,Q05.8,Q05.9,Q06.0,Q06.1,Q06.2,Q06.3,Q06.4,Q06.8,Q06.9,Q07.00,Q07.01,Q07.02,Q07.03,Q07.8,Q07.9,G11.0,G11.1,G11.2,G11.3,G11.4,G11.8,G11.9,G32.81,S14.0XXA,S14.0XXD,S14.0XXS,S14.101A,S14.101D,S14.101S,S14.102A,S14.102D,S14.102S,S14.103A,S14.103D,S14.103S,S14.104A,S14.104D,S14.104S,S14.105A,S14.105D,S14.105S,S14.106A,S14.106D,S14.106S,S14.107A,S14.107D,S14.107S,S14.108A,S14.108D,S14.108S,S14.109A,S14.109D,S14.109S,S14.121A,S14.121D,S14.121S,S14.122A,S14.122D,S14.122S,S14.123A,S14.123D,S14.123S,S14.124A,S14.124D,S14.124S,S14.125A,S14.125D,S14.125S,S14.126A,S14.126D,S14.126S,S14.127A,S14.127D,S14.127S,S14.128A,S14.128D,S14.128S,S14.129A,S14.129D,S14.129S,S14.131A,S14.131D,S14.131S,S14.132A,S14.132D,S14.132S,S14.133A,S14.133D,S14.133S,S14.134A,S14.134D,S14.134S,S14.135A,S14.135D,S14.135S,S14.136A,S14.136D,S14.136S,S14.137A,S14.137D,S14.137S,S14.138A,S14.138D,S14.138S,S14.139A,S14.139D,S14.139S,S14.141A,S14.141D,S14.141S,S14.142A,S14.142D,S14.142S,S14.143A,S14.143D,S14.143S,S14.144A,S14.144D,S14.144S,S14.145A,S14.145D,S14.145S,S14.146A,S14.146D,S14.146S,S14.147A,S14.147D,S14.147S,S14.148A,S14.148D,S14.148S,S14.149A,S14.149D,S14.149S,S14.151A,S14.151D,S14.151S,S14.152A,S14.152D,S14.152S,S14.153A,S14.153D,S14.153S,S14.154A,S14.154D,S14.154S,S14.155A,S14.155D,S14.155S,S14.156A,S14.156D,S14.156S,S14.157A,S14.157D,S14.157S,S14.158A,S14.158D,S14.158S,S14.159A,S14.159D,S14.159S,S24.0XXA,S24.0XXD,S24.0XXS,S24.101A,S24.101D,S24.101S,S24.102A,S24.102D,S24.102S,S24.103A,S24.103D,S24.103S,S24.104A,S24.104D,S24.104S,S24.109A,S24.109D,S24.109S,S24.131A,S24.131D,S24.131S,S24.132A,S24.132D,S24.132S,S24.133A,S24.133D,S24.133S,S24.134A,S24.134D,S24.134S,S24.139A,S24.139D,S24.139S,S24.141A,S24.141D,S24.141S,S24.142A,S24.142D,S24.142S,S24.143A,S24.143D,S24.143S,S24.144A,S24.144D,S24.144S,S24.149A,S24.149D,S24.149S,S24.151A,S24.151D,S24.151S,S24.152A,S24.152D,S24.152S,S24.153A,S24.153D,S24.153S,S24.154A,S24.154D,S24.154S,S24.159A,S24.159D,S24.159S,S34.01XA,S34.01XD,S34.01XS,S34.02XA,S34.02XD,S34.02XS,S34.101A,S34.101D,S34.101S,S34.102A,S34.102D,S34.102S,S34.103A,S34.103D,S34.103S,S34.104A,S34.104D,S34.104S,S34.105A,S34.105D,S34.105S,S34.109A,S34.109D,S34.109S,S34.111A,S34.111D,S34.111S,S34.112A,S34.112D,S34.112S,S34.113A,S34.113D,S34.113S,S34.114A,S34.114D,S34.114S,S34.115A,S34.115D,S34.115S,S34.119A,S34.119D,S34.119S,S34.121A,S34.121D,S34.121S,S34.122A,S34.122D,S34.122S,S34.123A,S34.123D,S34.123S,S34.124A,S34.124D,S34.124S,S34.125A,S34.125D,S34.125S,S34.129A,S34.129D,S34.129S,S34.131A,S34.131D,S34.131S,S34.132A,S34.132D,S34.132S,S34.139A,S34.139D,S34.139S,S34.3XXA
**Renal disease**	**HCC134:** T81.502A,T81.502D,T81.502S,T81.512A,T81.512D,T81.512S,T81.522A,T81.522D,T81.522S,T81.532A,T81.532D,T81.532S,T81.592A,T81.592D,T81.592S,T82.41XA,T82.41XD,T82.41XS,T82.42XA,T82.42XD,T82.42XS,T82.43XA,T82.43XD,T82.43XS,T82.49XA,T82.49XD,T82.49XS,T85.611A,T85.611D,T85.611S,T85.621A,T85.621D,T85.621S,T85.631A,T85.631D,T85.631S,T85.691A,T85.691D,T85.691S,T85.71XA,T85.71XD,T85.71XS,Y62.2,Z49.01,Z49.02,Z49.31,Z49.32,Z91.15,Z99.2
	**HCC135:** N17.0,N17.1,N17.2,N17.8,N17.9
	**HCC136 :** I12.0, I13.11, I13.2, N18.5, N18.6
	**HCC137:** N00.0,N00.1,N00.2,N00.3,N00.4,N00.5,N00.6,N00.7,N00.8,N00.9,N01.0,N01.1,N01.2,N01.3,N01.4,N01.5,N01.6,N01.7,N01.8,N01.9,N02.0,N02.1,N02.2,N02.3,N02.4,N02.5,N02.6,N02.7,N02.8,N02.9,N03.0,N03.1,N03.2,N03.3,N03.4,N03.5,N03.6,N03.7,N03.8,N03.9,N04.0,N04.1,N04.2,N04.3,N04.4,N04.5,N04.6,N04.7,N04.8,N04.9,N05.0,N05.1,N05.2,N05.3,N05.4,N05.5,N05.6,N05.7,N05.8,N05.9,N06.0,N06.1,N06.2,N06.3,N06.4,N06.5,N06.6,N06.7,N06.8,N06.9,N07.0,N07

© 2019 Ouayogodé MH et al. *JAMA Network Open.*

	.1,N07.2,N07.3,N07.4,N07.5,N07.6,N07.7,N07.8,N07.9,N08,N14.0,N14.1,N14.2,N14.3,N14.4,N15.0,N15.8,
**Peripheral vascular disease**	**HCC108:** I80.10,I80.11,I80.12,I80.13,I80.201,I80.202,I80.203,I80.209,I80.211,I80.212,I80.213,I80.219,I80.221,I80.222,I80.223,I80.229,I80.231,I80.232,I80.233,I80.239,I80.291,I80.292,I80.293,I80.299,I82.0,I82.210,I82.211,I82.220,I82.221,I82.290,I82.291,I82.3,I82.401,I82.402,I82.403,I82.409,I82.411,I82.412,I82.413,I82.419,I82.421,I82.422,I82.423,I82.429,I82.431,I82.432,I82.433,I82.439,I82.441,I82.442,I82.443,I82.449,I82.491,I82.492,I82.493,I82.499,I82.4Y1,I82.4Y2,I82.4Y3,I82.4Y9,I82.4Z1,I82.4Z2,I82.4Z3,I82.4Z9,I82.501,I82.502,I82.503,I82.509,I82.511,I82.512,I82.513,I82.519,I82.521,I82.522,I82.523,I82.529,I82.531,I82.532,I82.533,I82.539,I82.541,I82.542,I82.543,I82.549,I82.591,I82.592,I82.593,I82.599,I82.5Y1,I82.5Y2,I82.5Y3,I82.5Y9,I82.5Z1,I82.5Z2,I82.5Z3,I82.5Z9,I82.621,I82.622,I82.623,I82.629,I82.721,I82.722,I82.723,I82.729,I82.A11,I82.A12,I82.A13,I82.A19,I82.A21,I82.A22,I82.A23,I82.A29,I82.B11,I82.B12,I82.B13,I82.B19,I82.B21,I82.B22,I82.B23,I82.B29,I82.C11,I82.C12,I82.C13,I82.C19,I82.C21,I82.C22,I82.C23,I82.C29,E08.51,E09.51,E10.51,E11.51,E13.51,I70.0,I70.1,I70.201,I70.202,I70.203,I70.208,I70.209,I70.211,I70.212,I70.213,I70.218,I70.219,I70.221,I70.222,I70.223,I70.228,I70.229,I70.291,I70.292,I70.293,I70.298,I70.299,I70.301,I70.302,I70.303,I70.308,I70.309,I70.311,I70.312,I70.313,I70.318,I70.319,I70.321,I70.322,I70.323,I70.328,I70.329,I70.391,I70.392,I70.393,I70.398,I70.399,I70.401,I70.402,I70.403,I70.408,I70.409,I70.411,I70.412,I70.413,I70.418,I70.419,I70.421,I70.422,I70.423,I70.428,I70.429,I70.491,I70.492,I70.493,I70.498,I70.499,I70.501,I70.502,I70.503,I70.508,I70.509,I70.511,I70.512,I70.513,I70.518,I70.519,I70.521,I70.522,I70.523,I70.528,I70.529,I70.591,I70.592,I70.593,I70.598,I70.599,I70.601,I70.602,I70.603,I70.608,I70.609,I70.611,I70.612,I70.613,I70.618,I70.619,I70.621,I70.622,I70.623,I70.628,I70.629,I70.691,I70.692,I70.693,I70.698,I70.699,I70.701,I70.702,I70.703,I70.708,I70.709,I70.711,I70.712,I70.713,I70.718,I70.719,I70.721,I70.722,I70.723,I70.728,I70.729,I70.791,I70.792,I70.793,I70.798,I70.799,I70.92,I73.1,I73.81,I73.1,I73.1,I73.81,I73.89,I73.9,I791,I79.8,E08.52,E09.52,E10.52,E11.52,E13.52,I71.2,I71.4,I71.6,I71.9,I72.0,I72.1,I72.2,I72.3,I72.4,I72.8,I72.9,I77.0,I77.1,I77.2,I77.3,I77.4,I77.5,I77.6,I77.810,I77.811,I77.812,I77.819,I77.89,I77.9,I78.0,I79.0,K55.1,K55.8,K55.9,M31.8,M31.9
**Severe mental illness (SMI)**	**HCC57:** F20.0,F20.1,F20.2,F20.3,F20.5,F20.81,F20.89,F20.9,F25.0,F25.1,F25.8,F25.9
	**HCC58 :** F30.10,F30.11,F30.12,F30.13,F30.2,F30.3,F30.4,F30.8,F30.9,F31.0,F31.10,F31.11,F31.12,F31.13,F31.2,F31.30,F31.31,F31.32,F31.4,F31.5,F31.60,F31.61,F31.62,F31.63,F31.64,F31.70,F31.71,F31.72,F31.73,F31.74,F31.75,F31.76,F31.77,F31.78,F31.81,F31.89,F31.9,F34.8,F34.9,F39,F32.0,F32.1,F32.2,F32.3,F32.4,F32.5,F33.0,F33.1,F33.2,F33.3,F33.40,F33.41,F33.42,F33.8,F33.9,F22,F24,T14.91,T36.0X2A,T36.0X2S,T36.1X2A,T36.1X2S,T36.2X2A,T36.2X2S,T36.3X2A,T36.3X2S,T36.4X2A,T36.4X2S,T36.5X2A,T36.5X2S,T36.6X2A,T36.6X2S,T36.7X2A,T36.7X2S,T36.8X2A,T36.8X2S,T36.92XA,T36.92XS,T37.0X2A,T37.0X2S,T37.1X2A,T37.1X2S,T37.2X2A,T37.2X2S,T37.3X2A,T37.3X2S,T37.4X2A,T37.4X2S,T37.5X2A,T37.5X2S,T37.8X2A,T37.8X2S,T37.92XA,T37.92XS,T38.0X2A,T38.0X2S,T38.1X2A,T38.1X2S,T38.2X2A,T38.2X2S,T38.3X2A,T38.3X2S,T38.4X2A,T38.4X2S,T38.5X2A,T38.5X2S,T38.6X2A,T38.6X2S,T38.7X2A,T38.7X2S,T38.802A,T38.802S,T38.812A,T38.812S,T38.892A,T38.892S,T38.902A,T38.902S,T38.992A,T38.992S,T39.012A,T39.012S,T39.092A,T39.092S,T39.1X2A,T39.1X2S,T39.2X2A,T39.2X2S,T39.312A,T39.312S,T39.392A,T39.392S,T39.4X2A,T39.4X2S,T39.8X2A,T39.8X2S,T39.92XA,T39.92XS,T40.0X2A,T40.0X2S,T40.1X2A,T40.1X2S,T40.2X2A,T40.2X2S,T40.3X2A,T40.3X2S,T40.4X2A,T40.4X2S,T40.5X2A,T40.5X2S,T40.602A,T40.602S,T40.692A,T40.692S,T40.7X2A,T40.7X2S,T40.8X2A,T40.8X2S,T40.902A,T40.902S,T40.992A,T40.992S,T41.0X2A,T41.0X2S,T41.1X2A,T41.1X2S,T41.202A,T41.202S,T41.292A,T41.292S,T41.3X2A,T41.3X2S,T41.42XA,T41.42XS,T41.5X2A,T41.5X2S,T42.0X2A,T42.0X2S,T42.1X2A,T42.1X2S,T42.2X2A,T42.2X2S,T42.3X2A,T42.3X2S,T42.4X2A,T42.4X2S,T42.5X2A,T42.5X2S,T42.6X2A,T42.6X2S,T42.72XA,T42.72XS,T42.8X2A,T42.8X2S,T43.012A,T43.012S,T43.022A,T43.022S,T43.

© 2019 Ouayogodé MH et al. *JAMA Network Open.*

	1X2A,T43.1X2S,T43.202A,T43.202S,T43.212A,T43.212S,T43.222A,T43.222S,T43.292A,T43.292S,T43.3X2A,T43.3X2S,T43.4X2A,T43.4X2S,T43.502A,T43.502S,T43.592A,T43.592S,T43.602A,T43.602S,T43.612A,T43.612S,T43.622A,T43.622S,T43.632A,T43.632S,T43.692A,T43.692S,T43.8X2A,T43.8X2S,T43.92XA,T43.92XS,T44.0X2A,T44.0X2S,T44.1X2A,T44.1X2S,T44.2X2A,T44.2X2S,T44.3X2A,T44.3X2S,T44.4X2A,T44.4X2S,T44.5X2A,T44.5X2S,T44.6X2A,T44.6X2S,T44.7X2A,T44.7X2S,T44.8X2A,T44.8X2S,T44.902A,T44.902S,T44.992A,T44.992S,T45.0X2A,T45.0X2S,T45.1X2A,T45.1X2S,T45.2X2A,T45.2X2S,T45.3X2A,T45.3X2S,T45.4X2A,T45.4X2S,T45.512A,T45.512S,T45.522A,T45.522S,T45.602A,T45.602S,T45.612A,T45.612S,T45.622A,T45.622S,T45.692A,T45.692S,T45.7X2A,T45.7X2S,T45.8X2A,T45.8X2S,T45.92XA,T45.92XS,T46.0X2A,T46.0X2S,T46.1X2A,T46.1X2S,T46.2X2A,T46.2X2S,T46.3X2A,T46.3X2S,T46.4X2A,T46.4X2S,T46.5X2A,T46.5X2S,T46.6X2A,T46.6X2S,T46.7X2A,T46.7X2S,T46.8X2A,T46.8X2S,T46.902A,T46.902S,T46.992A,T46.992S,T47.0X2A,T47.0X2S,T47.1X2A,T47.1X2S,T47.2X2A,T47.2X2S,T47.3X2A,T47.3X2S,T47.4X2A,T47.4X2S,T47.5X2A,T47.5X2S,T47.6X2A,T47.6X2S,T47.7X2A,T47.7X2S,T47.8X2A,T47.8X2S,T47.92XA,T47.92XS,T48.0X2A,T48.0X2S,T48.1X2A,T48.1X2S,T48.202A,T48.202S,T48.292A,T48.292S,T48.3X2A,T48.3X2S,T48.4X2A,T48.4X2S,T48.5X2A,T48.5X2S,T48.6X2A,T48.6X2S,T48.902A,T48.902S,T48.992A,T48.992S,T49.0X2A,T49.0X2S,T49.1X2A,T49.1X2S,T49.2X2A,T49.2X2S,T49.3X2A,T49.3X2S,T49.4X2A,T49.4X2S,T49.5X2A,T49.5X2S,T49.6X2A,T49.6X2S,T49.7X2A,T49.7X2S,T49.8X2A,T49.8X2S,T49.92XA,T49.92XS,T50.0X2A,T50.0X2S,T50.1X2A,T50.1X2S,T50.2X2A,T50.2X2S,T50.3X2A,T50.3X2S,T50.4X2A,T50.4X2S,T50.5X2A,T50.5X2S,T50.6X2A,T50.6X2S,T50.7X2A,T50.7X2S,T50.8X2A,T50.8X2S,T50.902A,T50.902S,T50.992A,T50.992S,T50.A12A,T50.A12S,T50.A22A,T50.A22S,T50.A92A,T50.A92S,T50.B12A,T50.B12S,T50.B92A,T50.B92S,T50.Z12A,T50.Z12S,T50.Z92A,T50.Z92S,T51.0X2A,T51.0X2S,T51.1X2A,T51.1X2S,T51.2X2A,T51.2X2S,T51.3X2A,T51.3X2S,T51.8X2A,T51.8X2S,T51.92XA,T51.92XS,T52.0X2A,T52.0X2S,T52.1X2A,T52.1X2S,T52.2X2A,T52.2X2S,T52.3X2A,T52.3X2S,T52.4X2A,T52.4X2S,T52.8X2A,T52.8X2S,T52.92XA,T52.92XS,T53.0X2A,T53.0X2S,T53.1X2A,T53.1X2S,T53.2X2A,T53.2X2S,T53.3X2A,T53.3X2S,T53.4X2A,T53.4X2S,T53.5X2A,T53.5X2S,T53.6X2A,T53.6X2S,T53.7X2A,T53.7X2S,T53.92XA,T53.92XS,T54.0X2A,T54.0X2S,T54.1X2A,T54.1X2S,T54.2X2A,T54.2X2S,T54.3X2A,T54.3X2S,T54.92XA,T54.92XS,T55.0X2A,T55.0X2S,T55.1X2A,T55.1X2S,T56.0X2A,T56.0X2S,T56.1X2A,T56.1X2S,T56.2X2A,T56.2X2S,T56.3X2A,T56.3X2S,T56.4X2A,T56.4X2S,T56.5X2A,T56.5X2S,T56.6X2A,T56.6X2S,T56.7X2A,T56.7X2S,T56.812A,T56.812S,T56.892A,T56.892S,T56.92XA,T56.92XS,T57.0X2A,T57.0X2S,T57.1X2A,T57.1X2S,T57.2X2A,T57.2X2S,T57.3X2A,T57.3X2S,T57.8X2A,T57.8X2S,T57.92XA,T57.92XS,T58.02XA,T58.02XS,T58.12XA,T58.12XS,T58.2X2A,T58.2X2S,T58.8X2A,T58.8X2S,T58.92XA,T58.92XS,T59.0X2A,T59.0X2S,T59.1X2A,T59.1X2S,T59.2X2A,T59.2X2S,T59.3X2A,T59.3X2S,T59.4X2A,T59.4X2S,T59.5X2A,T59.5X2S,T59.6X2A,T59.6X2S,T59.7X2A,T59.7X2S,T59.812A,T59.812S,T59.892A,T59.892S,T59.92XA,T59.92XS,T60.0X2A,T60.0X2S,T60.1X2A,T60.1X2S,T60.2X2A,T60.2X2S,T60.3X2A,T60.3X2S,T60.4X2A,T60.4X2S,T60.8X2A,T60.8X2S,T60.92XA,T60.92XS,T61.02XA,T61.02XS,T61.12XA,T61.12XS,T61.772A,T61.772S,T61.782A,T61.782S,T61.8X2A,T61.8X2S,T61.92XA,T61.92XS,T62.0X2A,T62.0X2S,T62.1X2A,T62.1X2S,T62.2X2A,T62.2X2S,T62.8X2A,T62.8X2S,T62.92XA,T62.92XS,T63.002A,T63.002S,T63.012A,T63.012S,T63.022A,T63.022S,T63.032A,T63.032S,T63.042A,T63.042S,T63.062A,T63.062S,T63.072A,T63.072S,T63.082A,T63.082S,T63.092A,T63.092S,T63.112A,T63.112S,T63.122A,T63.122S,T63.192A,T63.192S,T63.2X2A,T63.2X2S,T63.302A,T63.302S,T63.312A,T63.312S,T63.322A,T63.322S,T63.332A,T63.332S,T63.392A,T63.392S,T63.412A,T63.412S,T63.422A,T63.422S,T63.432A,T63.432S,T63.442A,T63.442S,T63.452A,T63.452S,T63.462A,T63.462S,T63.482A,T63.482S,T63.512A,T63.512S,T63.592A,T63.592S,T63.612A,T63.612S,T63.622A,T63.622S,T63.632A,T63.632S,T63.692A,T63.692S,T63.712A,T63.712S,T63.792A,T63.792S,T63.812A,T63.812S,T63.822A,T63.822S,T63.832A,T63.832S,T63.892A,T63.892S,T63.92XA,T63.92XS,T64.02XA,T64.02XS,T64.82XA,T64.82XS,T65.0X2A,T65.0X2S,T65.1X2A,T65.1X2S,T65.212A,T65.212S,T65.222A,T65.222S,T65.292A,T65.292S,T65.3X2A,T65.3X2S,T65.4X2A,T65.4X2S,T65.5X2A,T65.5X2S,T65.6X2A,T65.6X2S,T65.812A,T65.812S,T65.822A,T65.822S,T65.832A,T65.832S,T65.892A,T65.892S,T65.92XA,T65.92XS,T71.112A,T71.112S,T71.122A,T71.122S,T71.132A,T71.132S,T71.152A,T71.152S,T71.162A,T71.162S,T71.192A,T71.192S,T71.222A,T71.222S,T71.232A,T71.232S,X71.0XXA,X71.0XXD,X71.0XXS,X71.1XXA,X71.1XXD,X71.1XXS,X71.2XXA,X71.2X

	XD,X71.2XXS,X71.3XXA,X71.3XXD,X71.3XXS,X71.8XXA,X71.8XXD,X71.8XXS,X71.9XXA,X71.9XXD,X71.9XXS,X72.XXXA,X72.XXXD,X72.XXXS,X73.0XXA,X73.0XXD,X73.0XXS,X73.1XXA,X73.1XXD,X73.1XXS,X73.2XXA,X73.2XXD,X73.2XXS,X73.8XXA,X73.8XXD,X73.8XXS,X73.9XXA,X73.9XXD,X73.9XXS,X74.01XA,X74.01XD,X74.01XS,X74.02XA,X74.02XD,X74.02XS,X74.09XA,X74.09XD,X74.09XS,X74.8XXA,X74.8XXD,X74.8XXS,X74.9XXA,X74.9XXD,X74.9XXS,X75.XXXA,X75.XXXD,X75.XXXS,X76.XXXA,X76.XXXD,X76.XXXS,X77.0XXA,X77.0XXD,X77.0XXS,X77.1XXA,X77.1XXD,X77.1XXS,X77.2XXA,X77.2XXD,X77.2XXS,X77.3XXA,X77.3XXD,X77.3XXS,X77.8XXA,X77.8XXD,X77.8XXS,X77.9XXA,X77.9XXD,X77.9XXS,X78.0XXA,X78.0XXD,X78.0XXS,X78.1XXA,X78.1XXD,X78.1XXS,X78.2XXA,X78.2XXD,X78.2XXS,X78.8XXA,X78.8XXD,X78.8XXS,X78.9XXA,X78.9XXD,X78.9XXS,X79.XXXA,X79.XXXD,X79.XXXS,X80.XXXA,X80.XXXD,X80.XXXS,X81.0XXA,X81.0XXD,X81.0XXS,X81.1XXA,X81.1XXD,X81.1XXS,X81.8XXA,X81.8XXD,X81.8XXS,X82.0XXA,X82.0XXD,X82.0XXS,X82.1XXA,X82.1XXD,X82.1XXS,X82.2XXA,X82.2XXD,X82.2XXS,X82.8XXA,X82.8XXD,X82.8XXS,X83.0XXA,X83.0XXD,X83.0XXS,X83.1XXA,X83.1XXD,X83.1XXS,X83.2XXA,X83.2XXD,X83.2XXS,X83.8XXA,X83.8XXD,X83.8XXS
**Substance use disorder (SUD)**	**HCC54:** F10.159,F10.231,10.232,F10.250,F10.251,F10.259,F10.26,F10.27,F10.950,F10.951,F10.959,F10.96,F10.97,F11.150,F11.151,F11.159,F11.250,F11.251,F11.259,F11.950,F11.951,F11.959,F12.150,F12.151,F12.159,F12.250,F12.251,F12.259,F12.950,F12.951,F12.959,F13.150,F13.151,F13.159,F13.231,F13.232,F13.250,F13.251,F13.259,F13.26,F13.27,F13.931,F13.932,F13.950,F13.951,F13.959,F13.96,F13.97,F14.150,F14.151,F14.159,F14.250,F14.251,F14.259,F14.950,F14.951,F14.959,F15.150,F15.151,F15.159,F15.250,F15.251,F15.259,F15.950,F15.951,F15.959,F16.150,F16.151,F16.159,F16.250,F16.251,F16.259,F16.950,F16.951,F16.959,F18.150,F18.151,F18.159,F18.17,F18.250,F18.251,F18.259,F18.27,F18.950,F18.951,F18.959,F18.97,F19.150,F19.151,F19.159,F19.16,F19.17,F19.232,F19.250,F19.251,F19.259,F19.26,F19.27,F19.931,F19.932,F19.950,F19.951,F19.959,F19.96,F19.97
	**HCC55:** F10.120,F10.121,F10.129,F10.14,F10.180,F10.181,F10.182,F10.188,F10.19,F10.20,F10.21,F10.220,F10.221,F10.229,F10.230,F10.239,F10.24,F10.280,F10.281,F10.288,F10.29,F10.920,F10.921,F10.929,F10.94,F10.980,F10.981,F10.982,F10.988,F10.99,F11.120,F11.121,F11.122,F11.129,F11.14,F11.181,F11.182,F11.189,F11.19,F11.20,F11.21,F11.220,F11.221,F11.222,F11.229,F11.23,F11.24,F11.281,F11.282,F11.288,F11.29,F11.920,F11.921,F11.922,F11.929,F11.93,F11.94,F11.981,F11.982,F11.988,F11.99,F12.120,F12.121,F12.122,F12.129,F12.180,F12.188,F12.19,F12.20,F12.21,F12.220,F12.221,F12.222,F12.229,F12.280,F12.288,F12.29,F12.920,F12.921,F12.922,F12.929,F12.980,F12.988,F12.99,F13.120,F13.121,F13.129,F13.14,F13.180,F13.181,F13.182,F13.188,F13.19,F13.20,F13.21,F13.220,F13.221,F13.229,F13.230,F13.239,F13.24,13.280,F13.281,F13.282,F13.288,F13.29,F13.920,F13.921,F13.929,F13.930,F13.939,F13.94,F13.980,F13.981,F13.982,F13.988,F13.99,F14.120,F14.121,F14.122,F14.129,F14.14,F14.180,F14.181,F14.182,F14.188,F14.19,F14.20,F14.21,F14.220,F14.221,F14.221,F14.229,F14.23,F14.24,F14.280,F14.281,F14.282,F14.288,F14.29,F14.920,F14.921,F14.922,F14.929,F14.94,F14.980,F14.981,F14.982,F14.988,F14.99,F15.120,F15.121,F15.122,F15.129,F15.14,F15.180,F15.181,F15.182,F15.188,F15.19,F15.20,F15.21,F15.220,F15.221,F15.222,F15.229,F15.23,F15.24,F15.280,F15.281,F15.282,F15.288,F15.29,F15.920,F15.921,F15.922,F15.929,F15.93,F15.94,F15.980,F15.981,F15.982,F15.988,F15.99,F16.120,F16.121,F16.122,F16.129,F16.14,F16.180,F16.183,F16.188,F16.19,F16.20,F16.21,F16.220,F16.221,F16.229,F16.24,F16.280,F16.283,F16.288,F16.29,F16.920,F16.921,F16.929,F16.94,F16.980,F16.983,F16.988,F16.99,F18.120,F18.121,F18.129,F18.14,F18.18,F18.19,F18.20,F18.21,F18.220,F18.221,F18.229,F18.24,F18.280,F18.288,F18.29,F18.920,F18.921,F18.929,F18.94,F18.980,F18.988,F18.99,F19.120,F19.121,F19.122,F19.129,F19.14,F19.180,F19.181,F19.182,F19.188,F19.19,F19.20,F19.21,F19.220,F19.221,F19.222,F19.229,F19.230,F19.239,F19.24,F19.280,F19.281,F19.282,F19.288,F19.29,F19.920,F19.921,F19.922,F19.929,F19.930,F19.939,F19.94,F19.980,F19.981,F19.982,F19.988,F19.9

© 2019 Ouayogodé MH et al. *JAMA Network Open.*

eTable 4. Description of provider number, Place of Service (POS) and Common Procedure Terminology (CPT) codes defining nursing facility utilization updated from Yun et al., (2010)

**Codes from Yun et al. 2010**	**Our Codes**	**Description**
	**Provider Number**	
	5000-6499	Skilled Nursing Facilities
**POS codes**		
31	31	Skilled Nursing facility
32	32	Nursing Facility
33	33	Custodial Care Facility
**CPT codes**		
	99304	Initial Nursing Facility Care
	99305	Initial Nursing Facility Care
	99306	Initial Nursing Facility Care
	99307	Subsequent Nursing Facility Care
	99308	Subsequent Nursing Facility Care
	99309	Subsequent Nursing Facility Care
	99310	Subsequent Nursing Facility Care
	99318	Other Nursing Facility Services
	99379	Physician Supervision of Nursing Facility Patient
	99380	Physician Supervision of Nursing Facility Patient
99301		Evaluation and management of a new or established patient involving an annual nursing facility assessment. Physicians typically spend 30 min at the bedside and on the patient’s facility floor or unit
99302		Evaluation and management of a new or established patient involving an annual nursing facility assessment; Physicians typically spend 40 min at the bedside and on the patient’s facility floor or unit
99303		Evaluation and management of a new or established patient involving an annual nursing facility assessment; Physicians typically spend 50 min at the bedside and on the patient’s facility floor or unit
99311		Subsequent nursing facility care, evaluation and management of a new or established patient; Physician usually spends 15 min at the bedside and on the patient’s facility floor or unit
99312		Subsequent nursing facility care, per day, for the evaluation management of a new or established patient physician usually spends 25 min at the bedside and on the patient’s facility floor or unit
99313		Subsequent nursing facility care, per day, for the evaluation and management of a new or established patient. Physician usually spends 35 min at the bedside and on the patient’s facility floor or unit
99315	99315	Nursing facility discharge day management; 30 min or less
99316	99316	Nursing facility discharge day management; more than 30 min

99379		Physician supervision of a nursing facility patient (patient not present)<br>requiring complex and multidisciplinary care, 15–29 min
99380		Physician supervision of a nursing facility patient (patient not present)<br>requiring complex and multidisciplinary care; 30 min or more
G0066		Physician supervision of a nursing facility patient (patient not present);<br>30 min or more per month

eTable 5. NSACO respondents versus non-respondent analysis: Descriptive characteristics of fee-for-service Medicare beneficiaries with complex needs attributed to ACO in 2016[†]

Characteristics	Attributed to MSSP ACO Not Reporting Care Management and Coordination of Complex Patients (Index = missing value) (n = 937 557)[a]	Attributed to MSSP ACO Reporting Care Management and Coordination of Complex Patients (n = 1 402 582)	P-value[ǂ]
**Cohort Entry**			
Frail Elderly (=1 if ≥2 Frailty Indicators),[b] No. (%)	197 918 (21.1)	301 476 (21.5)	<0.001
Multimorbidity (=1 if ≥2 Selected Chronic Conditions),[c] No. (%)	870 878 (92.9)	1 303 545 (92.9)	0.14
Frail Elderly and Multimorbidity (=1 if ≥2 Frailty Indicators & ≥2 Selected Chronic Conditions),[bc] No. (%)	131 239 (14.0)	202 439 (14.4)	<0.001
**Demographic Characteristics**			
Age, mean (SD), years	78.2 (8.0)	78.2 (8.0)	<0.001[Ᵽ]
Gender, No. (%)			
Female	520 669 (55.5)	773 102 (55.1)	<0.001
Race,[d] No. (%)			
Non-Hispanic white	744 002 (79.4)	1 207 758 (86.1)	<0.001
Black	99 022 (10.6)	105 184 (7.5)	<0.001
Hispanic	47 496 (5.1)	52 433 (3.7)	<0.001
Asian/Pacific Islander	33 884 (3.6)	17 962 (1.3)	<0.001
Other	13 153 (1.4)	19 245 (1.4)	0.05
Lives in High Poverty (>20%) census tract, No. (%)	171 985 (18.3)	238 987 (17.0)	<0.001
Dual Medicare and Medicaid Status, No. (%)	202 580 (21.6)	255 244 (18.1)	<0.001
Nursing Home Resident, No. (%)	267 051 (28.5)	415 338 (29.6)	<0.001
**Clinical Condition History**			
No. of Hierarchical Condition Categories (HCCs), Count, median (IQR)	3.0 (2.0 — 5.0)	3.0 (2.0 — 5.0)	0.11
Mortality, Death recorded in 2016, No. (%)	110 195 (11.8)	169 974 (12.1)	<0.001

eTable 5. NSACO respondents versus non-respondent analysis: Descriptive characteristics of fee-for-service Medicare beneficiaries with complex needs attributed to ACO in 2016 (Continued)†

**Outcomes**	Attributed to MSSP ACO Not Reporting Care Management and Coordination of Complex Patients (Index = missing value) (n = 937 557)[a]	Attributed to MSSP ACO Reporting Care Management and Coordination of Complex Patients (n = 1 402 582)	P-value‡
**Quality of Care, Count per Beneficiary, median (range)**			
PQI Admissions (All Cause)	0.0 (0.0 — 52.0)	0.0 (0.0 — 53.0)	<0.001
30-day Readmissions (All Cause)[e]	0.0 (0.0 — 4.0)	0.0 (0.0 — 5.0)	0.03
**Healthcare Utilization, Count per Beneficiary, median, (IQR)**			
E&M Visits in Ambulatory Settings	14.0 (8.0 — 21.0)	14.0 (8.0 — 21.0)	0.16
Acute Care/Critical Access Hospital Admissions	0.0 (0.0 — 1.0)	1.0 (0.0 — 1.0)	<0.001
Inpatient Days[e]	10.0 (3.0 — 31.0)	10.0 (4.0 — 31.0)	<0.001
ED Visits	1.0 (0.0 — 200.0)	1.0 (0.0 — 2.0)	<0.001
**Spending, $, median, (IQR)**			
Total Spending[f]	13987 (4727 — 35775)	14182 (4808 — 35820)	<0.001
PAC Spending[g]	0 (0 — 5374)	0 (0 — 5283)	<0.001
**Interactions with the Healthcare System, median (IQR)**			
Healthcare System Contact Days, Count	28.0 (17.0 — 43.0)	28.0 (17.0 — 44.0)	<0.001
Continuity of Care (Index) {0,1}[h]	0.13 (0.08 – 0.22)	0.12 (0.08 – 0.21)	<0.001

Abbreviations: ACO: accountable care organization, MSSP: Medicare shared savings program, NSACO: national survey of accountable care organizations, IQR: interquartile range, PQI: prevention quality indicator, E&M: evaluation and management, ED: emergency department, PAC: post-acute care

†Includes beneficiaries age 66 years and older. ‡T-test means comparison test. ᵖDifferences in means were in higher decimals. [a] Non-MSSP ACOs include all provider organizations without an MSSP contract. [b] Frailty indicators included Abnormality of Gait, Malnutrition/Abnormal Loss of Weight and Underweight, Adult Failure to Thrive, Cachexia, Debility, Fall, Muscular Wasting and Disuse Atrophy, Muscle Weakness, Decubitus Ulcer of Skin/Pressure Ulcer, Senility Without Mention of Psychosis, Malaise and Fatigue, Durable Medical Equipment Use, Nursing or Personal Care Services. [c] Selected chronic conditions included Coronary Artery Disease (CAD), Cancer, Connective Tissue Disorders, Congestive Heart Failure (CHF), Diabetes, Dementia, Chronic Obstructive Pulmonary Disease (COPD), Hematologic/Thrombotic Disease, HIV/AIDS, Immune Disease, Liver Disease, Parkinson's/Huntington's, Paralysis, Peripheral Vascular Disease, Renal Disease, Cerebral Hemorrhage/Stroke, Severe Mental Illness, Substance Use Disorder. [d] Race/ethnicity percentages may not sum to 100 percent due to rounding error. [e] Inpatient days are only computed for patients with observable inpatient admissions. Readmissions were only reported for beneficiaries with non-zero inpatient days. [f] Total spending included MedPAR (inpatient), carrier (physician/supplier), outpatient facility, hospice, durable medical equipment (DME), and home health. [g] PAC spending included settings such as skilled nursing facilities, home health agency, inpatient rehabilitation facility, long-term care hospital, outpatient rehabilitation facility and comprehensive

outpatient rehabilitation facility. [h] Continuity of care indexes were only calculated for a subset of beneficiaries with four or more visits in 2016.

© 2019 Ouayogodé MH et al. *JAMA Network Open.*

eTable 6. Association between ACO Intensity of Care Management and Coordination Index and Outcomes for Fee-for-Service Medicare Beneficiaries with Complex Needs, 2016 (n = 1 402 582)†

	**Regression Model Coefficient (95% CI) ǂ**			
	**Covariate-Adjusted Ᵽᵼ**		**Covariate-Adjustedᵽᵼ**	
**Outcome Variables**	ACO Care Management/ Coordination Index 2nd Tertile	ACO Care Management/ Coordination Index 2nd Tertile	ACO Care Management/ Coordination Index 2nd Tertile	ACO Care Management/ Coordination Index 3rd (Top) Tertile
**<u>Quality of Care</u>**				
Prevention Quality Indicator (PQI) Admissions (All Cause) per 100 Beneficiaries	-0.52 (-1.76-0.73)	-0.14 (-1.38-1.09)	-0.31 (-1.10-0.49)	-0.16 (-0.96-0.64)
30-day Readmissions (All Cause) per 100 Beneficiaries	0.07 (-0.46-0.60)	-0.08 (-0.66-0.50)	0.01 (-0.42-0.45)	0.002 (-0.48-0.48)
**<u>Healthcare Utilization</u>**				
Evaluation and Management (E&M) Visits in Ambulatory Settings per 100 Beneficiaries	-60.46 (-176.58-55.66)	-13.07 (-138.38-112.23)	-49.57 (-136.03-36.90)	-15.18 (-126.14-95.78)
Acute Care/Critical Access Hospital Admissions per 100 Beneficiaries	-0.13 (-4.18-3.93)	-0.97 (-4.70-2.76)	0.22 (-2.73-3.18)	-0.40 (-3.03-2.23)
Inpatient Days	-0.28 (-1.18-0.62)	-0.10 (-1.19-0.99)	-0.23 (-0.93-0.46)	-0.16 (-1.03-0.72)
Emergency Department (ED) Visits per 100 Beneficiaries	1.61 (-5.17-8.40)	-1.09 (-8.27-6.10)	2.14 (-2.26-6.55)	-0.73 (-2.24-4.78)
**<u>Spending</u>**				
Total Spending	-229 (-2065-1607)	-850 (-2567-867)	-97 (-1319-1124)	-369 (-1617-878)
Post-Acute Care (PAC) Spending	-439 (-1158-280)	-355 (-1119-409)	-265 (-714-183)	-227 (-767-313)

**Interactions with the Healthcare System**				
Healthcare System Contact Days	-1.31 (-3.31-0.69)	-0.86 (-2.82-1.10)	-1.20 (-2.58-0.19)	-0.66 (-2.37-1.05)
Continuity of Care (Index)	-0.005 (-0.01-0.002)	-0.004 (-0.01-0.004)	-0.005 (-0.01-0.002)	-0.004 (-0.01-0.004)

Abbreviations: ACO: accountable care organization, MSSP: Medicare shared savings program, PQI: prevention quality indicator, E&M: evaluation and management, ED: emergency department, PAC: post-acute care
†Includes beneficiaries age 66 years and older. ǂ Least Squares (LS) regressions were estimated for all outcome variables and regression coefficients with the associated 95% confidence intervals were reported. Heteroscedasticity robust Standard errors (not reported) were clustered at the ACO level. Each model represents a single regression for each outcome variable and all ACO Care Management/Coordination index tertiles are jointly estimated. ᵽ Regressions were adjusted for cohort entry flags (frail elderly and an interaction term for being both frail and having multiple chronic conditions), demographics (age, sex, race and ethnicity group, high poverty status), dual eligibility for Medicaid status, and nursing home residency. Regression coefficients on covariates are available upon request. ŧ The sample was restricted to include frail elderly (>=2 frailty indicators) and those with at least 3 of the selected 18 chronic conditions (n=820 328). ŧ Census regions effects were included (n=1 402 582)

© 2019 Ouayogodé MH et al. *JAMA Network Open.*
